# A methodology for the structural and functional analysis of signaling and regulatory networks

**DOI:** 10.1186/1471-2105-7-56

**Published:** 2006-02-07

**Authors:** Steffen Klamt, Julio Saez-Rodriguez, Jonathan A Lindquist, Luca Simeoni, Ernst D Gilles

**Affiliations:** 1 Max-Planck Institute for Dynamics of Complex Technical Systems, Sandtorstrasse 1, D-39106 Magdeburg, Germany; 2 Institute for Immunology, University of Magdeburg, Leipziger Strasse 44, D-39120 Magdeburg, Germany

## Abstract

**Background:**

Structural analysis of cellular interaction networks contributes to a deeper understanding of network-wide interdependencies, causal relationships, and basic functional capabilities. While the structural analysis of metabolic networks is a well-established field, similar methodologies have been scarcely developed and applied to signaling and regulatory networks.

**Results:**

We propose formalisms and methods, relying on adapted and partially newly introduced approaches, which facilitate a structural analysis of signaling and regulatory networks with focus on functional aspects. We use two different formalisms to represent and analyze interaction networks: interaction graphs and (logical) interaction hypergraphs. We show that, in interaction graphs, the determination of feedback cycles and of all the signaling paths between any pair of species is equivalent to the computation of elementary modes known from metabolic networks. Knowledge on the set of signaling paths and feedback loops facilitates the computation of intervention strategies and the classification of compounds into activators, inhibitors, ambivalent factors, and non-affecting factors with respect to a certain species. In some cases, qualitative effects induced by perturbations can be unambiguously predicted from the network scheme. Interaction graphs however, are not able to capture AND relationships which do frequently occur in interaction networks. The consequent logical concatenation of all the arcs pointing into a species leads to Boolean networks. For a Boolean representation of cellular interaction networks we propose a formalism based on logical (or signed) interaction hypergraphs, which facilitates in particular a logical steady state analysis (LSSA). LSSA enables studies on the logical processing of signals and the identification of optimal intervention points (targets) in cellular networks. LSSA also reveals network regions whose parametrization and initial states are crucial for the dynamic behavior.

We have implemented these methods in our software tool *CellNetAnalyzer *(successor of *FluxAnalyzer*) and illustrate their applicability using a logical model of T-Cell receptor signaling providing non-intuitive results regarding feedback loops, essential elements, and (logical) signal processing upon different stimuli.

**Conclusion:**

The methods and formalisms we propose herein are another step towards the comprehensive functional analysis of cellular interaction networks. Their potential, shown on a realistic T-cell signaling model, makes them a promising tool.

## Background

Evolution has equipped cells with exquisite signaling systems which allow them to sense their environment, receive and process signals in a hierarchically organized manner and to react accordingly [1]. The complexity of the corresponding molecular machineries, in accordance with the complicated tasks they have to perform, is overwhelming. In the last few years, as a key element to the growing popularity of systems biology, mathematical tools have been applied to the analysis of signaling data [2]. Ordinary differential equations relying on kinetic descriptions of the underlying molecular interactions are arguably the most used approach for modeling signaling networks (e.g. [3-6]). A number of theoretical methods have been devised and employed for the reconstruction (reverse engineering) of signaling or, more generally, interaction networks (which may represent signaling but also other types or abstractions of cellular networks such as genetic regulatory networks) based on perturbation experiments [7]. The approaches rely on methods ranging from Bayesian networks (e.g. [8]) to metabolic control analysis [9,10].

Relatively few methods have been proposed so far for analyzing the structure of a *given *signaling (or any interaction) network. This is somewhat surprising since structural analysis of metabolic networks is a well-established field and proved to be successful to recognize relationships between structure, function, and regulation of metabolic networks [11]. Structural analysis will be particularly useful in large signaling networks, where a simple visual inspection is not possible and at the same time the construction of precise quantitative models is practically infeasible due to the huge amount of required, but generally unknown, kinetic parameters and concentration values. However, the reconstruction of large signaling networks is still in its first stages [2,12].

Structural or qualitative approaches that have been employed for interaction networks include statistical large-scale analyses in protein-protein networks (e.g. [13]). These studies are important for examining statistical properties of the interaction graph and for understanding its global organization but they provide relatively few insights into the function of the network. Papin and co-workers [14,15] were the first to adapt methods from the constraint-based approach (frequently used for structural analysis of metabolic networks [11]) to analyze stoichiometric models of signaling pathways. Recently, graph-theoretical descriptions of signaling networks have been examined [16-18]. Finally, Boolean networks as discrete approximations of quantitative models have been used for logical analyses of small signaling networks e.g. [19]. However, the majority of studies relying on the Boolean approach deal with genetic interaction networks, many of which have a relatively small size (ca. 10 species; e.g. [20,21]), however, recently more complicated networks have also been investigated [22,23].

In this contribution, we propose formalisms for representing signaling and other interaction networks mathematically and present a collection of methods facilitating structural analysis of the respective network models. Rather than introducing completely new concepts, we will systematize and adapt existing formalisms and methods, often motivated from structural analyses of metabolic networks, towards a *functional *analysis of the structure of a signaling network. Issues that can be addressed with the proposed methods include:

• check of the plausibility and consistency of the network structure

• identification of all or particular signaling pathways, feedback loops and crosstalks

• network-wide functional interdependencies between network elements

• identification of the different modes of (logical) input/output behavior

• predicting responses (phenotypes) after changes in network structure

• finding targets and intervention points in the network for repressing or provoking a certain behavior or response

• analysis of structural network properties like redundancy and robustness

Structural analysis is not based on quantitative and dynamic properties and can thus only provide qualitative answers. However, some insights into the dynamic properties can nevertheless often be obtained, because fundamental properties of the dynamic behavior are often governed by the network structure [24]. While we will focus on signaling networks, the methods can be easily applied to any kind of interaction network, including gene regulatory systems. Apart from a toy model, we will exemplify our methods on a model of signaling pathways in T-cells.

## Results and discussion

### Mass and signal flows in cellular interaction networks

The reader familiar to the structural analysis of stoichiometric networks may notice that, in the case of metabolic networks, many of the issues in the task list of the previous section have been handled by the constraint-based approach [11]. For example, the identification of functional pathways and studying the input (substrates)/output (products) behavior of stoichiometric reaction networks is facilitated by elementary-modes analysis [25,26]. Flux Balance Analysis is another related technique often used for phenotype predictions of metabolic mutants [11,27]. Recently, the concept of minimal cut sets has been introduced for identifying targets in metabolic networks [28,29]. Therefore, it seems reasonable to apply these methods to signaling networks. However, some fundamental differences in the way the network elements interact may complicate a direct transfer:

(1) The constraint-based framework assumes steady-state, while in signaling networks a transient behavior can often be observed. (However, as will be discussed below, many useful insights of signaling networks can be obtained from using a static approach.)

(2) In stoichiometric networks, any arrow (reaction) leading from educts to products can be seen as an "activating" (producing) connection for the products. Therefore, employing stoichiometric framework it is difficult or only indirectly possible to express an inhibitory action of a species onto another.

(3) Probably the most significant difference is that the edges (i.e. the connections between the species) in metabolic networks carry flows of *mass *whereas edges in signaling networks may carry mass *and/or *information (signal) flow. Of course, at the molecular level, any interaction between species in the cell can be written as a stoichiometric equation. However, whereas mass flow is connected to a real consumption of participating compounds, signal flow is usually characterized by a recycling of certain species (e.g. enzymes) so that these species can mediate the signal transfer continuously (until they are degraded).

**Figure 1:**
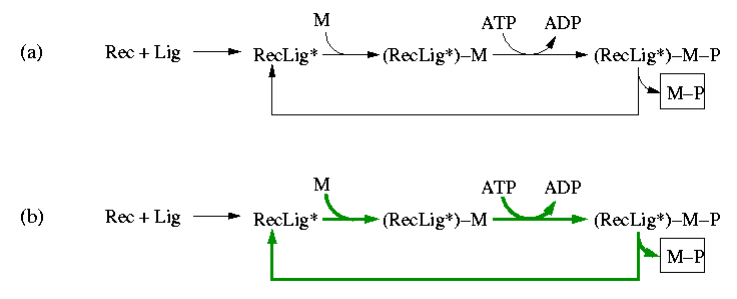
(a) Example of a typical signaling pathway where mass and signal flow occur simultaneosly (Rec = receptor; Lig = ligand; RecLig* = (active) receptor-ligand-complex; M = molecule; M-P = phosphorylated molecule M). (b) The (only) elementary mode in this example which follows when M, M-P, ADP, ATP, Rec and Lig are considered as external (boundary) species. The involved reactions are indicated by green, thick arrows. In its net stoichiometry, this elementary mode converts M and ATP into M-P and ADP, whereas RecLig* is recycled in the overall process. Importantly, the mandatory process of building the receptor-ligand-complex RecLig* (hence, the causal dependeny of M-P from the availability of Rec and Lig) is not reflected by this mode.

A typical example, namely the activation of a receptor tyrosine kinase (Figure 1(a)) [30], illustrates the simultaneous occurrence of mass and signal flow. A ligand (Lig) binds to the extracellular domain of a receptor (Rec) yielding a receptor-ligand complex which can undergo further changes (e.g. by autophosphorylation or/and dimerization). We denote the outcome by RecLig*. This complex is now able to phosphorylate another molecule (M). Accordingly, M binds to RecLig* and becomes phosphorylated (M-P) by the expense of ATP. At the end, M-P is released, recycling also the activated receptor-ligand complex RecLig*.

The first step in this scheme can be considered as a mass flow. However, the cycle in which RecLig* phosphorylates M, is a mass flow with respect to M and ATP, but a signaling flow with respect to RecLig*, as the latter is indeed required for driving this cycle but not consumed (because recycled) in the overall stoichiometry.

In performing a structural analysis we are interested in extracting signaling paths from the network scheme. Therefore, it may seem reasonable to compute elementary modes, which typically represent pathways in reaction networks with mass flow [25]. A basic property of elementary modes is that the (relative) mass flow represented by an elementary mode keeps the "internal" species in a balanced state. Internal species (here: RecLig*, RecLig*-M, RecLig*-M-P) are within the system's boundary, whereas the external species (here: Rec, Lig, M, M-P, ADP, ATP) are considered as pools which are balanced by processes lying outside the system's boundaries. Computing the elementary modes from the respective stoichiometric model of Figure 1(a) gives exactly one mode which reflects the discussed role of RecLig* as a kinase (Figure 1(b)): in its net stoichiometry, this elementary mode converts the external species M and ATP into M-P and ADP, whereas RecLig* is recycled. Since RecLig* is neither consumed nor produced in the overall process, the first step (building the receptor-ligand complex) is not involved in this mode simply because a continuous synthesis of RecLig* would lead to an accumulation of this species, which is inconsistent with the steady-state assumption of elementary modes. Thus, the causal dependency of M-P from the availability of Rec and Lig is not reflected by the mass flow concept of elementary modes. Note that exactly the same conceptual problem would arise when enzymes and enzyme synthesis would be considered explicitly in stoichiometric studies of metabolic networks.

**Figure 2:**
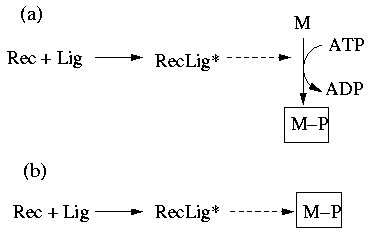
Interpretation of Figure 1 as an interaction network.

The example demonstrates that we require a framework with the ability to account for mass *and *signal flows. Handling both mass and signal flows formally equivalent as *interactions *could be a suitable approach. Interpreting Figure 1(a)) as a diagram of interactions we could redraw it as depicted in Figure 2(a). The dashed arrow indicates that RecLig* catalyzes the phosphorylation of M to M-P. If we assume that ADP, ATP, and M are always present, we get the simple chain shown in Figure 2(b) expressing that Rec and Lig are required to obtain RecLig* (or to activate RecLig*), and that RecLig* is required to get M-P. If we do not further distinguish between the two types of arrows and thus consider mass and signal flows as formally equivalent, the causal connections between the species would, nevertheless, still be captured correctly. This abstract representation of different types of interactions will thus be used herein.

The following two sections will deal first with interaction *graphs *and later with the more general (logical) interaction *hypergraphs*. The basic difference between these two related approaches can be illustrated by how they deal with a connection such as "Rec + Lig" in Figure 2(b). If we interpret it as "Rec activates RecLig* and Lig activates RecLig*" then the concept of *interaction graphs *is applicable (discussed in the following section). However, it would be more accurate to say that "Rec AND Lig are required *simultaneously *for building RecLig*", and it is this more refined approach that leads to the concept of *interaction hypergraphs*, which will be discussed in further details later on.

### Analyzing interaction graphs

#### Definition of interaction graphs

Interaction (or causal influence) graphs are frequently used to show direct dependencies between species in signaling, genetic, or protein-protein interaction networks. The nodes in these graphs may represent, depending on the network type and the level of abstraction, receptors, ligands, effectors, kinases, genes, transcription factors, metabolites, proteins, and other compounds, while each edge describes a relation between *two *of these species. In signaling and gene regulatory networks, two further characteristics are usually specified for each edge: a *direction *(which species influences which) and a *sign *("+" or "- ", depending on whether the influence is activating (level increasing) or inhibiting (level decreasing)). Formally, we represent a directed interaction or causal influence graph as a signed directed graph ***G ***= (***V***, ***A***), where ***V ***is the set of vertices or nodes (species) and ***A ***the set of labeled directed edges [31,32]. Directed edges are usually called arcs and an arc from vertex *i *(*tail*) to *j *(*head*) is denoted by an ordered tuple {*i*,*j*,*s*} with *i, j *∈ ***V ***and *s *∈ {+,- }.

Sometimes, for example in protein-protein interaction networks, the directions of the edges remain unspecified. We will not consider such undirected interaction graphs explicitly, however, many of the issues discussed in the following can be transferred to undirected graphs (e.g. by representing an undirected edge by two (forward and backward) arcs).

The structure of a signed graph can be stored conveniently by an *m *x *q *incidence matrix **B **in which the columns correspond to the *q *arcs (interactions) and the rows to the *m *nodes (species), similar as in stoichiometric matrices of metabolic reaction networks [33]. For the *k*-th arc {*i, j, s*} a (-1) is stored in the *k*-th column of **B **for the tail vertex (*i*) and (+1) for the head vertex (*j*) of arc *k*. Hence, **B**_*i*,*k *_= -1 and **B**_*j*,*k *_= 1 and **B**_*l*,*k *_= 0 (*l*≠ *i, j*). For storing the signs, a *q*-vector **s **is introduced whose *k*-th element is (+1) if arc *k *is positive and (-1) if *k *is negative.

Self-loops (arcs connecting a species with itself) are not considered here but could be stored in a separate list since they would appear as a zero column in the incidence matrix.

Note that, as far as the memory requirement is concerned, the structure of a graph can be stored more efficiently than by an incidence matrix, e.g. by using adjacency lists [34]. However, since we will present methods directly operating on the incidence matrix, we refer herein to this representation.

Signal transduction networks are usually characterized by an input, intermediate, and output layer (cf. [16]). The input domain consists only of species having no predecessor, which can thus not be activated from other species in the graph. Such *sources *(typical representatives are receptors and ligands) are starting points of signal transduction pathways and can easily be identified from the incidence matrix since their corresponding row contains no positive entry. In contrast, the output layer consists only of nodes having no successor. These *sinks*, usually corresponding to transcription factors or genes, are identifiable as rows in **B **which have no negative entry. The set of source and sink nodes define the boundaries of the network under investigation. They play here a similar role as the external metabolites in stoichiometric studies [33]. The intermediate layer functions as the actual signal transduction and processing unit. It consists of the intermediate species, all of which have at least one predecessor and at least one successor, i.e. they are influenced and they influence other elements. Such species contain both -1 and +1 entries in the incidence matrix. In reconstructed signaling networks, the detection of all sink and source species may help to detect gaps in the network, e.g. when a species should be an intermediate but is classified as a sink or source.

The presence of sinks and sources are a consequence of setting borders to the system of interest. Sometimes there are no sinks or/and no sources, especially in models of gene regulatory networks (see e.g. the networks studied in [21]), but this does not impose limitations to the approaches presented here.

**Figure 3:**
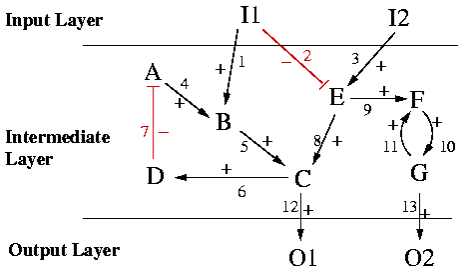
Example of a directed interaction graph (TOYNET). Arcs 2 and 7 indicate inhibiting interactions, while all others are activating.

A toy example of a (directed) interaction graph that will serve for illustrations throughout this paper is given in Figure 3. This interaction graph, called TOYNET, consists of two sources (I1, I2), two sinks (O1, O2), 7 intermediate species (A,..., G), two inhibiting (arcs 2 and 7) and 11 activating interactions. Incidence matrix **B **of TOYNET reads (the sign vector **s **is given on the top of **B**):

B=+−++++−++++++(−1−10000000000000−10000000000000−10010000001001−10000000000001−101000−10000001−10000000110000−1−10000000000001−11000000000001−10−100000000000100000000000001)I1I2ABCDEFGO1O2     (1)
 MathType@MTEF@5@5@+=feaafiart1ev1aaatCvAUfeBSjuyZL2yd9gzLbvyNv2CaerbuLwBLnhiov2DGi1BTfMBaeXatLxBI9gBaerbbjxAHXgarqqtubsr4rNCHbGeaGqik81rpy0dbbc9akVeYhH8F4riQ9vqqrFD0p0PFj0xirFj0dXdbba90m6JbbG8FasPpaIOFHK8Feey0dXdarVALq=f0=yqaiVgqr=qN+vr0=vr0=vqpWqaaeaabiGaciaacaqabeaadaqabqaaaOqaamXvP5wqonvsaeHbhL2B2fMBULgic92BRbacfeGae8NqaiKaeyypa0tbaeqabiGaaaqaauaabeqab0aaaaaaaeaacWaGasv=aaGHRaWkaeaacWaGaYC=aaGHsislaeaacWaGasK=aaGHRaWkaeaacWaGaIP=aaGHRaWkaeaacWaGaIV=aaGHRaWkaeaacWaiaA4kaScabaGaeyOeI0cabaGamaiGqcaaay4kaScabaGamaiGGeaaay4kaScabaGamaiGagaaay4kaScabaGamaiG4haaay4kaScabaGamaiG8jaaay4kaScabaGamaiGqlaaay4kaScaaaqaaaqaamaabmaabaqbaeqabSqdaaaaaaaaaeaacqGHsisliuaacqGFXaqmaeaacqGHsislcqGFXaqmaeaacqGFWaamaeaacqGFWaamaeaacqGFWaamaeaacqGFWaamaeaacqGFWaamaeaacqGFWaamaeaacqGFWaamaeaacqGFWaamaeaacqGFWaamaeaacqGFWaamaeaacqGFWaamaeaacqGFWaamaeaacqGFWaamaeaacqGHsislcqGFXaqmaeaacqGFWaamaeaacqGFWaamaeaacqGFWaamaeaacqGFWaamaeaacqGFWaamaeaacqGFWaamaeaacqGFWaamaeaacqGFWaamaeaacqGFWaamaeaacqGFWaamaeaacqGFWaamaeaacqGFWaamaeaacqGFWaamaeaacqGHsislcqGFXaqmaeaacqGFWaamaeaacqGFWaamaeaacaaIXaaabaGae4hmaadabaGae4hmaadabaGae4hmaadabaGae4hmaadabaGae4hmaadabaGae4hmaadabaGaaGymaaqaaiab+bdaWaqaaiab+bdaWaqaaiab+fdaXaqaaiabgkHiTiab+fdaXaqaaiab+bdaWaqaaiab+bdaWaqaaiab+bdaWaqaaiab+bdaWaqaaiab+bdaWaqaaiab+bdaWaqaaiab+bdaWaqaaiab+bdaWaqaaiab+bdaWaqaaiab+bdaWaqaaiab+bdaWaqaaiab+bdaWaqaaiab+fdaXaqaaiabgkHiTiab+fdaXaqaaiab+bdaWaqaaiaaigdaaeaacqGFWaamaeaacqGFWaamaeaacqGFWaamaeaacqGHsislcqGFXaqmaeaacqGFWaamaeaacqGFWaamaeaacqGFWaamaeaacqGFWaamaeaacqGFWaamaeaacqGFWaamaeaacqGFXaqmaeaacqGHsislcqGFXaqmaeaacqGFWaamaeaacqGFWaamaeaacqGFWaamaeaacqGFWaamaeaacqGFWaamaeaacqGFWaamaeaacqGFWaamaeaacqGFXaqmaeaacqGFXaqmaeaacqGFWaamaeaacqGFWaamaeaacqGFWaamaeaacqGFWaamaeaacqGHsislcqGFXaqmaeaacqGHsislcqGFXaqmaeaacqGFWaamaeaacqGFWaamaeaacqGFWaamaeaacqGFWaamaeaacqGFWaamaeaacqGFWaamaeaacqGFWaamaeaacqGFWaamaeaacqGFWaamaeaacqGFWaamaeaacqGFWaamaeaacqGFWaamaeaacqGFXaqmaeaacqGHsislcqGFXaqmaeaacqGFXaqmaeaacqGFWaamaeaacqGFWaamaeaacqGFWaamaeaacqGFWaamaeaacqGFWaamaeaacqGFWaamaeaacqGFWaamaeaacqGFWaamaeaacqGFWaamaeaacqGFWaamaeaacqGFWaamaeaacqGFXaqmaeaacqGHsislcqGFXaqmaeaacqGFWaamaeaacqGHsislcqGFXaqmaeaacqGFWaamaeaacqGFWaamaeaacqGFWaamaeaacqGFWaamaeaacqGFWaamaeaacqGFWaamaeaacqGFWaamaeaacqGFWaamaeaacqGFWaamaeaacqGFWaamaeaacqGFWaamaeaacqGFXaqmaeaacqGFWaamaeaacqGFWaamaeaacqGFWaamaeaacqGFWaamaeaacqGFWaamaeaacqGFWaamaeaacqGFWaamaeaacqGFWaamaeaacqGFWaamaeaacqGFWaamaeaacqGFWaamaeaacqGFWaamaeaacqGFWaamaeaacqGFXaqmaaaacaGLOaGaayzkaaaabaqbaeqabSqaaaaaaeaacqGFjbqscqGFXaqmaeaacqGFjbqscqGFYaGmaeaacqGFbbqqaeaacqGFcbGqaeaacqGFdbWqaeaacqGFebaraeaacqGFfbqraeaacqGFgbGraeaacqGFhbWraeaacqGFpbWtcqGFXaqmaeaacqGFpbWtcqGFYaGmaaaaaiaaxMaacaWLjaGaaiikaiab+fdaXiaacMcaaaa@0668@    

#### Identification of feedback loops

Even though some analysis methods (e.g. Bayesian networks) rely on acyclic networks where feedbacks are not allowed, one of the most important features of signaling and regulatory networks are their feedback loops [3,5,18,21,35-38]. Positive feedbacks are responsible and even required [39] for multiple steady state behavior in dynamical systems. In biological systems, multistationarity plays a central role in differentiation processes and for epigenetic and switch-like behavior. In contrast, negative feedback loops are essential for homeostatic mechanisms (i.e. for adjusting and maintaining levels of system variables) or for generating oscillatory behavior [35].

Most reports demonstrating the role and consequences of feedback loops analyze relatively small networks where the cycles can be easily recognized from the network scheme but rather few works address the question of how feedback cycles can be identified systematically. This is particularly important in large interaction graphs, where a detection by simple visual inspection is impossible, especially when feedback loops overlap.

A feedback loop is, in graph theory, a *directed cycle *or *circuit*. A circuit is defined as a sequence *C = *{**a**_1_,...,**a**_*w*_} of arcs that starts and ends at the same vertex *k *and visits (with the exception of *k*) no vertex twice, i.e. *C = *{**a**_1_,...,**a**_*w*_} = {{*k*, *l*_1_}, {*l*_1_,*l*_2_},..., {*l*_*w*-1_,*k*}} such that all nodes *k*, *l*_1_, *l*_2 _... *l*_*w*-1 _are distinct. The parity of the number of negative signs of the arcs in *C *determines whether the feedback loop is negative (odd number of negative signs) or positive (even). In the example TOYNET two feedback loops can be found: (i) the arc sequence {4,5,6,7} which is negative (since one negative arc (7) is involved), and (ii) the sequence {10,11}, which is positive (because the signs of both arcs in this circuit are positive). Obviously, sinks and sources (and all arcs connected to these nodes) can never be involved in any circuit.

Computing all directed cycles in large graphs is computationally a difficult task. Algorithms that can be found in the literature usually rely on backtracking strategies (e.g. [16,40]). Here, we introduce a different approach where the circuits are identified as elementary modes establishing a direct link to metabolic network analysis. Circuits can be formally represented by a *q*-vector **c **in which *c*_*i *_= 1 if arc *i *is involved in the circuit and *c*_*i *_= 0 otherwise. A circuit vector fulfills the equation

**B c **= **0 **   (2)

and hence, lies in the null space of the incidence matrix of the graph [32,41]. Generally, any vector **c **obeying (2) fulfills a so-called conservation law and is called a *circulation *which may be envisioned as a flow cycling around in the network [42]. Eq. (2) is strongly related to the mass balance equation of metabolic networks in steady state. In fact, considering the graph as a reaction network with the arcs being irreversible mono-molecular reactions, the incidence matrix would be equivalent to the stoichiometric matrix and any circulation would be equivalent to a stationary flux distribution. Note that not all circulations are circuits: the linear combinations of circuit vectors do also yield circulations but are not (elementary) circuits. Precisely, circuits are special circulations having two additional properties. First, they must be admissible with respect to the directions of the involved arcs, i.e. only non-negative values are allowed for **c**:

*c*_*i*_≥ 0 for all *i*.     (3)

Second, circuits are *non-decomposable *circulations, i.e. the set of arcs building up the circuit **c**, expressed by P(**c**) = {*i*: *c*_*i *_> 0}, is irreducible:

There is no non-zero vector **d **fulfilling eqs. (2) and (3) and P(**d**)⊂ P(**c**)    (4)

Eqs. (2) and (3) and condition (4) close the complete analogy to elementary modes. In fact, cycles or circuits are the elementary modes in the special case of graphs (elementary modes are defined for *any *matrix in eq. (2), not only for the very special shape of incidence matrices related to graphs). Any feasible stationary flux vector in a metabolic network can be obtained by non-negative linear combinations of elementary modes. Equivalently, any circulation vector can be decomposed into a non-negative linear combination of circuit vectors. Note that, multiplying a (circuit) vector **c**, that fulfills (2)-(4), by a scalar *b*>0 yields another vector **v **= *b***c **which represents the same circuit because the same arcs compose it (are unequal to zero). Moreover, all non-zero components in a circuit vector are equal to each other. Therefore we can always normalize the vector in such a way that we obtain the binary representative of this circuit where all components are either "1" or "0".

In metabolic networks, elementary modes reveal not only internal cycles but also, even with higher relevance, metabolic pathways connecting input and output species. Continuing with the analogy to interaction graphs, in the next subsection we will see that elementary modes can be used to identify not only feedback loops but also signaling paths.

#### Signaling (influence) paths between two species

When the interaction graph is very large it becomes difficult to see whether a species S1 can influence (activate or inhibit) another species S2 and via which distinct pathways this can happen. Computing the complete set of directed paths between a given pair (S1, S2) of species is therefore often desirable. A path *P *= {**a**_1_,...,**a**_*w*_} is, similarly to a feedback circuit, a sequence of arcs where none of the nodes is visited more than once, but in the case of a signaling path the start node S1 is distinct from the end node S2, i.e. *P = *{**a**_1_,...,**a**_*w*_} = {{*S1*,*l*_1_}, {*l*_1_,*l*_2_}, ..., {*l*_*w*-1_,*S2*}} such that all nodes *S1, S2*, *l*_1_, *l*_2 _... *l*_*w*-1 _are distinct.

**Figure 4:**
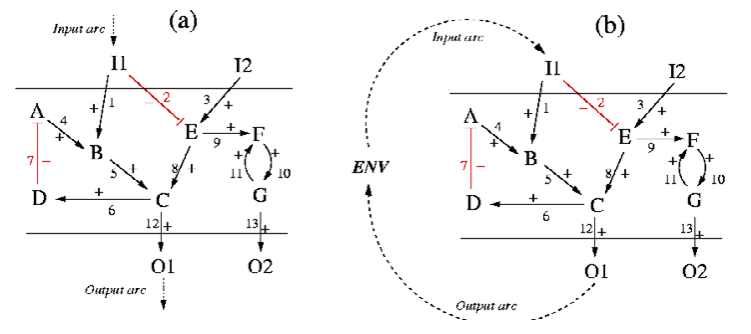
Computation of all signaling paths between two species (here: between I1 and O1). (a) via the incorporation of a "simplified" input and output arc; (b) with explicit introduction of an ENV („environment") node. Computing the elementary modes from the respective incidence matrix for (a) and (b) yields basically the same result, namely all paths between I1 and O1, as well as the two feedback circuits in the intermediate layer.

To obtain the signaling pathways from S1 to S2 we proceed as follows (Figure 4(a)): we add an "input arc" for S1 (i.e. a new column in the incidence matrix **B **containing only zeros except a (+1) for S1) and an "output arc" for S2 (another new column in **B **containing only zeros except a (-1) for S2. Then, computation of the elementary modes in this network will provide the original feedback loops without participation of the input and the output arc (as shown above) and additionally all paths starting with the input arc at S1 and ending with the output arc at S2, with the latter revealing all possible routes between S1 and S2.

Admittedly, the introduced input and output arcs have no tail or no head, respectively, and would therefore not be edges in the graph-theoretical sense, but this has no consequence for the analysis described within this contribution. In fact, this procedure is equivalent to adding in the incidence matrix a "dummy" node representing the environment (ENV), an "input arc" from ENV to S1 and an "output arc" from S2 to ENV (Figure 4(b)). Computing the elementary modes from the resulting incidence matrix would produce the feedback circuits as well as the circuits running over ENV. The latter represent the paths leading from S1 to S2. In the procedure described above ENV is simply removed from the incidence matrix leading to the same results.

In order to obtain only the paths from S1 to S2 (without the feedback loops), one can enforce the input and output arc to be involved by using an extension of the algorithm for computing elementary modes [43].

**Figure 5:**
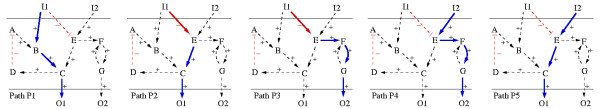
All signaling paths linking the input layer (source species) with the output layer (sink species) in TOYNET.

Furthermore, we may also add several input and output edges simultaneously. For example, if we are interested in all the paths connecting the input layer with the output layer, i.e. all routes leading from a source to a sink node, we add to each source an input edge and to each sink an output edge and compute the elementary modes (and, optionally, discard the feedback circuits where neither a source nor a sink participates). In this way we obtain the same set of signaling paths as if the elementary modes would be computed separately for each possible pair of source and sink nodes. Figure 5 shows the complete set of signaling paths connecting the input with the output layer of TOYNET.

Analogously to the feedback loops, we assign to each signaling path an "overall sign" indicating whether A activates (+) or inhibts (-) B along this path. Again, the parity of the signs of the arcs in the path determine whether the influence is positive (even number of negative signs) or negative (odd number of signs).

To sum up, feedback loops and influence paths in interaction graphs can be identified as elementary modes (or, equivalently, as extreme rays of convex cones [44]) from the respective incidence matrix. Similar conclusions have recently been drawn by Xiong et al. [45], albeit the authors computed paths only between sink and source nodes and only within unsigned graphs (i.e. they did not consider inhibitory effects). Feedback circuits were also not considered. Hence, here we extend and generalize those results.

The equivalence of signaling paths and loops to elementary modes allows one the advantage to use the highly optimized algorithms for computing elementary modes [43,44,46].

#### Combinatorial studies on signaling paths

The computation of all paths between a pair of species helps us to recognize all the different ways in which a signal can propagate between two nodes. In metabolic pathway analysis, a statistical or combinatorial analysis of the participation and co-occurrences of reactions in elementary modes proved to be useful for obtaining system-wide properties, such as the detection of essential reactions/enzymes or correlated reaction sets (enzyme subsets) [11,26,47].

In principle, similar features are of interest also for signaling paths and feedback loops. However, two important issues arise in interaction graphs that require a special treatment. First, we have two different types of pathways, positives and negatives. Owing to their opposite meanings we often need to analyze them separately in statistical assessments. Second, in metabolic networks we are particularly interested in the reactions (edges), because they correspond to enzymes that are subject to regulatory processes and can be knocked-out in experiments. In contrast, in interaction graphs we are usually more interested in the nodes, since they are often knocked-out in experiments or medical treatments, either via mutations, siRNA or by specific inhibitors. An edge in signaling networks represents mostly a direct interaction between a pair of species and has therefore no mediator. In some cases, an edge can directly be targeted by e.g. a mutation at the corresponding binding site of one of the two nodes species involved. Here, we will focus on species participation, albeit similar computations can be made for the edges.

As mentioned several times, in signaling networks we are often interested in all the different ways by which a certain transcription factor (or any other species from the output layer) can be activated or inhibited by signals arriving the input layer. For this purpose, we compute all signaling paths leading from source nodes located in the input layer down to a certain sink species *s *of interest. We denote the set of all these paths by ***I***_*s*_, which can be dissected in the two disjoint subsets of activating and inhibiting paths: ***I ***= Is+
 MathType@MTEF@5@5@+=feaafiart1ev1aaatCvAUfKttLearuWrP9MDH5MBPbIqV92AaeXatLxBI9gBaebbnrfifHhDYfgasaacH8akY=wiFfYdH8Gipec8Eeeu0xXdbba9frFj0=OqFfea0dXdd9vqai=hGuQ8kuc9pgc9s8qqaq=dirpe0xb9q8qiLsFr0=vr0=vr0dc8meaabaqaciaacaGaaeqabaqabeGadaaakeaaieWacqWFjbqsdaqhaaWcbaGaem4CamhabaGaey4kaScaaaaa@304D@ ⋃ Is−
 MathType@MTEF@5@5@+=feaafiart1ev1aaatCvAUfKttLearuWrP9MDH5MBPbIqV92AaeXatLxBI9gBaebbnrfifHhDYfgasaacH8akY=wiFfYdH8Gipec8Eeeu0xXdbba9frFj0=OqFfea0dXdd9vqai=hGuQ8kuc9pgc9s8qqaq=dirpe0xb9q8qiLsFr0=vr0=vr0dc8meaabaqaciaacaGaaeqabaqabeGadaaakeaaieWacqWFjbqsdaqhaaWcbaGaem4CamhabaGaeyOeI0caaaaa@3058@. Each source species *i *can then be classified into one of the following four influence classes with respect to *s*:

(1) activator of *s *(*i *is involved in at least one path of Is+
 MathType@MTEF@5@5@+=feaafiart1ev1aaatCvAUfKttLearuWrP9MDH5MBPbIqV92AaeXatLxBI9gBaebbnrfifHhDYfgasaacH8akY=wiFfYdH8Gipec8Eeeu0xXdbba9frFj0=OqFfea0dXdd9vqai=hGuQ8kuc9pgc9s8qqaq=dirpe0xb9q8qiLsFr0=vr0=vr0dc8meaabaqaciaacaGaaeqabaqabeGadaaakeaaieWacqWFjbqsdaqhaaWcbaGaem4CamhabaGaey4kaScaaaaa@304D@ and in no path of Is−
 MathType@MTEF@5@5@+=feaafiart1ev1aaatCvAUfKttLearuWrP9MDH5MBPbIqV92AaeXatLxBI9gBaebbnrfifHhDYfgasaacH8akY=wiFfYdH8Gipec8Eeeu0xXdbba9frFj0=OqFfea0dXdd9vqai=hGuQ8kuc9pgc9s8qqaq=dirpe0xb9q8qiLsFr0=vr0=vr0dc8meaabaqaciaacaGaaeqabaqabeGadaaakeaaieWacqWFjbqsdaqhaaWcbaGaem4CamhabaGaeyOeI0caaaaa@3058@)

(2) inhibitor of *s *(*i *is involved in at least one path of Is−
 MathType@MTEF@5@5@+=feaafiart1ev1aaatCvAUfKttLearuWrP9MDH5MBPbIqV92AaeXatLxBI9gBaebbnrfifHhDYfgasaacH8akY=wiFfYdH8Gipec8Eeeu0xXdbba9frFj0=OqFfea0dXdd9vqai=hGuQ8kuc9pgc9s8qqaq=dirpe0xb9q8qiLsFr0=vr0=vr0dc8meaabaqaciaacaGaaeqabaqabeGadaaakeaaieWacqWFjbqsdaqhaaWcbaGaem4CamhabaGaeyOeI0caaaaa@3058@ and in no path of Is+
 MathType@MTEF@5@5@+=feaafiart1ev1aaatCvAUfKttLearuWrP9MDH5MBPbIqV92AaeXatLxBI9gBaebbnrfifHhDYfgasaacH8akY=wiFfYdH8Gipec8Eeeu0xXdbba9frFj0=OqFfea0dXdd9vqai=hGuQ8kuc9pgc9s8qqaq=dirpe0xb9q8qiLsFr0=vr0=vr0dc8meaabaqaciaacaGaaeqabaqabeGadaaakeaaieWacqWFjbqsdaqhaaWcbaGaem4CamhabaGaey4kaScaaaaa@304D@)

(3) ambivalent factor for *s *(*i *is involved in at least one (inhibiting) path of Is−
 MathType@MTEF@5@5@+=feaafiart1ev1aaatCvAUfKttLearuWrP9MDH5MBPbIqV92AaeXatLxBI9gBaebbnrfifHhDYfgasaacH8akY=wiFfYdH8Gipec8Eeeu0xXdbba9frFj0=OqFfea0dXdd9vqai=hGuQ8kuc9pgc9s8qqaq=dirpe0xb9q8qiLsFr0=vr0=vr0dc8meaabaqaciaacaGaaeqabaqabeGadaaakeaaieWacqWFjbqsdaqhaaWcbaGaem4CamhabaGaeyOeI0caaaaa@3058@ and in at least one (activating) path of Is+
 MathType@MTEF@5@5@+=feaafiart1ev1aaatCvAUfKttLearuWrP9MDH5MBPbIqV92AaeXatLxBI9gBaebbnrfifHhDYfgasaacH8akY=wiFfYdH8Gipec8Eeeu0xXdbba9frFj0=OqFfea0dXdd9vqai=hGuQ8kuc9pgc9s8qqaq=dirpe0xb9q8qiLsFr0=vr0=vr0dc8meaabaqaciaacaGaaeqabaqabeGadaaakeaaieWacqWFjbqsdaqhaaWcbaGaem4CamhabaGaey4kaScaaaaa@304D@)

(4) without any influence on *s *(*i *is not involved in any path of ***I***_*s*_)

In TOYNET, we see from Figure 5 that I2 is a pure activator and I1 an ambivalent factor for O1. With respect to O2, I1 is an inhibitor and I2 again an activator. The qualitative response of *s *after perturbing the level of a non-affecting species, or of an inhibitor or activator can be predicted unambiguously (namely unchanged or decreasing or increasing, respectively) as long as the network has no negative feedback loop. Negative feedback loops limit such qualitative predictions for activators (or inhibitors): if there is any path from an activator (inhibitor) to *s *that touches a negative feedback loop (i.e. at least one species on the path is involved in a negative feedback) then the resulting effect in perturbation experiments can not be predicted uniquely (cf. [36]). This case occurs in TOYNET for I2 with respect to O1: I2 is an activator of O1 but the only connecting path (P5 in Figure 5) goes through species C which participates in the negative feedback circuit. Thus, although at least a transient increase in O1 can be expected after up-regulating I2, we cannot exclude that the negative feedback drives the level of O1 below its initial level at a certain time point after increasing the level of I2. We therefore call an activator (inhibitor) *p *of *s *a *total activator *(*total inhibitor*) of *s *if there is no path from *p *to a species in a negative feedback circuit that is in turn connected to *s*.

Positive feedbacks do not limit these qualitative up/down-predictions because they cannot change the monotone effect of the respective input signal, e.g. when increasing the level of I2 in TOYNET we can expect an increase in the level of O2 after some time.

To summarize, regarding the influence of a species *p *on another species *s *we have 6 possible cases: total and non-total activator, total and non-total inhibitor, ambivalent factor and non-influencing species. Note that, by computing the connecting signaling paths, this classification procedure can be applied not only between a source and a sink node but also between any pair of species, e.g. between a source and an intermediate, an intermediate and a sink, and two intermediates. In TOYNET, for example, *F *is a total activator of O2 and has no influence on O1, whereas D is an inhibitor but not a total one of O1 because it is connected to (even involved in) a negative feedback circuit.

Additionally, as the complement of incoming paths, we can also determine the paths starting in a certain species *s *showing us which nodes and arcs are reachable from (and influenceable by) *s*. As a further generalization, sets of incoming and/or outgoing paths can also be defined not only for a single species *s *but also for a set ***S ***of species. This might be useful, for example, when we are interested in all paths ending (starting) in a certain subset of the sink (source) nodes.

Investigations of influence and signaling paths as proposed above provide, apart from pair-pair relationships (e.g. "*a *is a (total) activator of *b" *or "*a *has no influence on *b*"), global properties (e.g. *a *is a (total) activator of all sink species). Some other useful structural features and constraints can be detected by a statistical or combinatorial analysis of certain path sets (partially, similar ideas have been proposed by [14] for stoichiometric models of signaling networks):

• Essential species (arcs): When focussing on a specific signaling event, e.g. the activation of a certain species by signals from the input layer, we may identify essential species (or arcs) with respect to this event. For example, species E and arc 9 are essential for activating O2 but non-essential for the activating paths leading to O1 in TOYNET.

• Species (arc) participation: A more quantitative measure can be obtained by giving percentages of all those activating and/or inhibiting pathways, in which the species or arc is involved. One may only relate the relative participation to the paths where the respective species or arc is involved or to the complete set of paths. For example, I2 is involved in 50% of all positive paths coming from the input layer and activating O1, while I2 is involved in 100% of all paths activating O2 (but only 50% of the paths coming from I2 lead to O2). Arc 9 is involved in one activating and one inhibiting path leading to O2. Thus, only 50% of the paths running over this arc are activating, however, it is involved in all (100%) activating paths connecting sources with O2. Similar considerations can be done regarding feedback loops: in TOYNET, species D and A as well as arcs 6, 7 and 11 are not involved in paths connecting input with output layers and have thus a special importance in establishing the negative (D, A, arcs 6 and 7) and positive (arc 11) feedback. (Note that a similar measure for the importance of a species or arc is betweenness centrality [48]. This importance measure is well-known in graph theory and checks how many *shortest *paths between pairs of nodes are running over the respective node or arc.)

• Redundancy: The total number of paths activating (inhibiting) a species is a measure for the redundancy in the system.

• Path length: The length distribution of signaling paths provides a rough idea on the compactness of the network [18].

• Crosstalk: Using our framework, crosstalk might be defined as a place (node) where paths from different source nodes cross each other for the first time. For example, E is a crosstalk species in TOYNET (signals of I1 and I2 cross) whereas F and G are not. In some cases, however, crosstalk is a more complex phenomenon where different nodes are involved. For example, at species C a path coming from I1 via B and another path from I2 via E meet each other. However, I1 and I2 have also met earlier in E and, additionally, the action of I1 on C via B is already influenced by I2 in species B since I2 can act on B via the path visiting E, C, D and A.

#### Distance matrix and dependency matrix

Some applications presented in this section require exhaustive enumerations of signaling paths becoming computationally challenging in large networks. However, in some cases we only want to know whether *any *activating and/or *any *inhibiting path between two nodes exists or whether there is any positive or any negative feedback circuit in which a certain species is involved. For such "existence questions" we can often apply standard methods from graph theory. A very useful object is the distance matrix **D **which can be obtained with low computational demand by computing the shortest distances (shortest path lengths) between each pair of species (e.g. Dijkstra's algorithm [32]). **D **has dimension *m *× *m *and the element **D**_*ij *_stores the length of the shortest path for traveling from node *i *to node *j*, being **D**_*ij *_= ∞ if no paths exists between *i *and *j*. The distance matrix shows immediately

• which elements can be influenced by species *i *(the *i*-th row of **D**)

• which nodes can influence species *i *(*i*-th column of **D**)

• whether feedback circuits exist: if the distance **D**_*ii *_from a node *i *back to itself is finite, then *i *is involved in at least one feedback loop. Furthermore, if **D**_*ij *_and the transposed element **D**_*ji *_are finite, **D**_*ij*_, **D**_*ji*_≠∞, then a feedback between species *i *and *j *exists.

By an extension of the usual shortest path algorithm (not shown), we may also compute separately a matrix **D**^pos ^for the shortest *positive *paths and another **D**^neg ^for the shortest *negative *paths. Table 1 shows the distance matrices **D**^pos ^and **D**^neg ^from TOYNET.

**Table 1:** Shortest length of positive/negative paths in TOYNET (∞= no path exists). Values in the diagonal indicate whether the respective element is involved in a positive/negative feedback loop. See also the dependency matrix in Figure 6.

	I1	I2	A	B	C	D	E	F	G	O1	O2
I1	∞/∞	∞/∞	4/4	1/∞	2/2	3/3	∞/1	∞/2	∞/3	3/3	∞/4
I2	∞/∞	∞/∞	∞/4	∞/5	2/∞	3/∞	1/∞	2/∞	3/∞	3/∞	4/∞
A	∞/∞	∞/∞	∞/4	1/∞	2/∞	3/∞	∞/∞	∞/∞	∞/∞	3/∞	∞/∞
B	∞/∞	∞/∞	∞/3	∞/4	1/∞	2/∞	∞/∞	∞/∞	∞/∞	2/∞	∞/∞
C	∞/∞	∞/∞	∞/2	∞/3	∞/4	1/∞	∞/∞	∞/∞	∞/∞	1/∞	∞/∞
D	∞/∞	∞/∞	∞/1	∞/2	∞/3	∞/4	∞/∞	∞/∞	∞/∞	∞/4	∞/∞
E	∞/∞	∞/∞	∞/3	∞/4	1/∞	2/∞	∞/∞	1/∞	2/∞	2/∞	3/∞
F	∞/∞	∞/∞	∞/∞	∞/∞	∞/∞	∞/∞	∞/∞	2/∞	1/∞	∞/∞	2/∞
G	∞/∞	∞/∞	∞/∞	∞/∞	∞/∞	∞/∞	∞/∞	1/∞	2/∞	∞/∞	1/∞
O1	∞/∞	∞/∞	∞/∞	∞/∞	∞/∞	∞/∞	∞/∞	∞/∞	∞/∞	∞/∞	∞/∞
O2	∞/∞	∞/∞	∞/∞	∞/∞	∞/∞	∞/∞	∞/∞	∞/∞	∞/∞	∞/∞	∞/∞

Note that by taking the minimum values from **D**^pos ^and **D**^neg^, **D **can be obtained. Moreover, the two matrices **D**^pos ^and **D**^neg^, whose computation is reasonably possible in very large networks, are sufficient to classify all species into (total/non-total) activators, (total/non-total) inhibitors, ambivalent factors, and non-influencing nodes with respect to a certain compound *y*. The reason is that this classification requires only knowledge on the *existence *of positive and negative paths between species pairs and on the *existence *of negative feedback loops. For example, a species *x *is a total activator of *y *if (i) at least one positive path from *x *to *y *exits (Dx,ypos
 MathType@MTEF@5@5@+=feaafiart1ev1aaatCvAUfKttLearuWrP9MDH5MBPbIqV92AaeXatLxBI9gBaebbnrfifHhDYfgasaacH8akY=wiFfYdH8Gipec8Eeeu0xXdbba9frFj0=OqFfea0dXdd9vqai=hGuQ8kuc9pgc9s8qqaq=dirpe0xb9q8qiLsFr0=vr0=vr0dc8meaabaqaciaacaGaaeqabaqabeGadaaakeaaieqacqWFebardaqhaaWcbaGaemiEaGNaeiilaWIaemyEaKhabaGaemiCaaNaem4Ba8Maem4Camhaaaaa@3603@ ≠ ∞) and if (ii) no negative path from *x *to *y *exists (Dx,yneg
 MathType@MTEF@5@5@+=feaafiart1ev1aaatCvAUfKttLearuWrP9MDH5MBPbIqV92AaeXatLxBI9gBaebbnrfifHhDYfgasaacH8akY=wiFfYdH8Gipec8Eeeu0xXdbba9frFj0=OqFfea0dXdd9vqai=hGuQ8kuc9pgc9s8qqaq=dirpe0xb9q8qiLsFr0=vr0=vr0dc8meaabaqaciaacaGaaeqabaqabeGadaaakeaaieqacqWFebardaqhaaWcbaGaemiEaGNaeiilaWIaemyEaKhabaGaemOBa4MaemyzauMaem4zaCgaaaaa@35D3@ = ∞) and if (iii) for any species *z *that is influenced by *x *(**D**_*x, z *_≠ ∞) and connected to *y *(**D**_*z, y *_≠ ∞) it holds, that *z *is not involved in a negative feedback (Dz,zneg
 MathType@MTEF@5@5@+=feaafiart1ev1aaatCvAUfKttLearuWrP9MDH5MBPbIqV92AaeXatLxBI9gBaebbnrfifHhDYfgasaacH8akY=wiFfYdH8Gipec8Eeeu0xXdbba9frFj0=OqFfea0dXdd9vqai=hGuQ8kuc9pgc9s8qqaq=dirpe0xb9q8qiLsFr0=vr0=vr0dc8meaabaqaciaacaGaaeqabaqabeGadaaakeaaieqacqWFebardaqhaaWcbaGaemOEaONaeiilaWIaemOEaOhabaGaemOBa4MaemyzauMaem4zaCgaaaaa@35D9@ = ∞).

**Figure 6:**
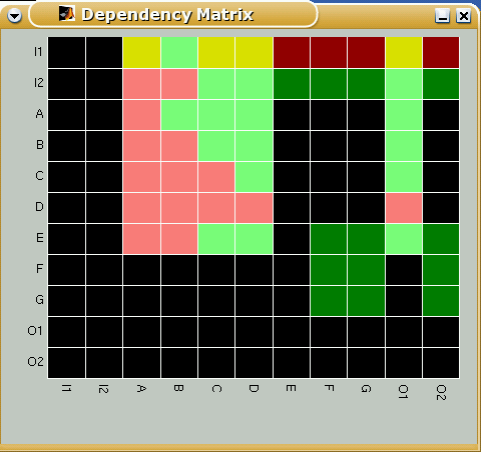
Dependency matrix of TOYNET. The color of a matrix element **M**_*xy *_has the following meaning: (i) dark green: x is an total activator of y; (ii) light green: x is a (non-total) activator of y; (iii) dark red: x is a total inhibitor of y; (iv) light red: x is a (non-total) inhibitor of y; (v) yellow: x is an ambivalent factor for y; (vi) black: x does not influence y;

For representing species dependencies in a compact manner, we introduce the *dependency matrix ***M, **which shows all the pair-wise dependencies, e.g. by using 6 different colors (for the 6 possible cases). Thereby, the color of matrix element **M**_*xy *_indicates whether species *x *is a total/non-total activator or a total/non-total inhibitor or an ambivalent factor or a non-influencing node for species *y*. Again, *x *= *y *is allowed, indicating feedbacks. Figure 6 shows the dependency matrix for TOYNET.

Although the distance and dependency matrices store a wealth of structural information in a very condensed manner, some applications still require a full enumeration of all available signaling paths. One case is the systematic determination of minimal cut sets.

#### Minimal cut and intervention sets in interaction graphs

Searching for intervention strategies in signaling networks is of high relevance in experimental and, in particular, medical applications. Recently, the concept of *minimal cut sets *has been introduced, which facilitates the identification of efficient intervention strategies (cuts) and, at the same time, the recognition of potential failure modes in a given biochemical reaction network [28,29]. Basically, in the most general version, a minimal cut set (MCS) is defined as a minimal (irreducible, non-decomposable) set of cuts (or failures) of edges or/and nodes that represses a certain functionality or behavior in the system [29]. For example, assume we want to prevent the activation of the sink node O1 in TOYNET. By removing nodes {B, E} one can be sure that an activation of O1 by an external stimulus becomes infeasible. The set {B, E} would thus be a cut set for preventing the activation of O1. Moreover, it is minimal since neither the removal of only B nor the removal of only E can guarantee that the "inhibition task" is achieved. Another minimal cut set would be {C}. C is thus essential for activating O1, as would be confirmed by participation analysis of all paths activating O1. A general algorithmic scheme for a systematic enumeration of MCSs in stoichiometric networks was given in [29]:

(i) Define a *deletion task*

(ii) Compute all minimal functional units (elementary modes) and specify the set of *target modes *that have to be attacked in order to achieve the deletion task

(iii) Compute the so-called *minimal hitting sets *of the target modes

We could proceed here in a similar way. First, a deletion task specifying the goal of our intervention is defined. In our example, the deletion task is "Prevent the activation of O1 by any external input". Hence, the signaling paths from the input layer to O1 are computed, which are P1, P2, and P5 (see Figure 5). However, according to our deletion task, the target set comprises only the paths P1 and P5, because only these two activate O1. Finally, the minimal hitting sets of the target paths have to be computed, which are the MCSs [26,29]. When cutting species, a hitting set ***T ***is a set of species that "hits" all target paths in a minimal way, i.e. for each target path there is at least one species that is contained in ***T ***and in the path. To be a *minimal *hitting set, no proper subset of ***T ***fulfills the hitting set condition. The minimal hitting sets of the target paths and hence the MCSs of our deletion task would be: {C}, {B, E}, {I2, B}, {I1, E} and {I1, I2}. Deletion tasks may be more complicated: for example, in TOYNET we might be interested to repress the activation of O1 *and *O2. Accordingly, the target paths would increase by one (P4 in Figure 5) resulting in another set of MCSs.

This example might suggest that we can use the same procedure as in metabolic networks, namely computing the minimal hitting sets with respect to the target paths. This naive approach works indeed for the case where the target paths do only involve positive arcs (as in our example). It can also be applied for interrupting any set of feedback circuits. For example, removing {A} interrupts the negative feedback circuit and deleting {D, F} interrupts both feedback circuits in TOYNET. However, in general, negatively signed arcs occurring in interaction graphs require a special treatment. Even the following simple activating path leading from a source species I to a sink species O contains pitfalls:

I→+A→−B→−C→+O
 MathType@MTEF@5@5@+=feaafiart1ev1aaatCvAUfKttLearuWrP9MDH5MBPbIqV92AaeXatLxBI9gBaebbnrfifHhDYfgasaacH8akY=wiFfYdH8Gipec8Eeeu0xXdbba9frFj0=OqFfea0dXdd9vqai=hGuQ8kuc9pgc9s8qqaq=dirpe0xb9q8qiLsFr0=vr0=vr0dc8meaabaqaciaacaGaaeqabaqabeGadaaakeaacqqGjbqsdaGdKaWcbaGaey4kaScabeGccaGLsgcacqqGbbqqdaGdKaWcbaGaeyOeI0cabeGccaGLsgcacqqGcbGqdaGdKaWcbaGaeyOeI0cabeGccaGLsgcacqqGdbWqdaGdKaWcbaGaey4kaScabeGccaGLsgcacqqGpbWtaaa@3C09@. If the activation of O is to be repressed, the signal flow along this path must be interrupted. Removal of one species in the chain should be sufficient. However, not all nodes are allowed to be cut. If species B is removed, its negative action on C would be interrupted, enabling in turn C to activate O. The reason is that B, according to the definitions, is an inhibitor of O and is therefore not a proper cut candidate. In fact, we could *add *(constitutively provide or activate) B to stop an activation of O. Generally, for attacking an *activating *path, only the species that have an activating effect on the end node of this path are proper cut candidates, whereas species inhibiting the end node should instead be kept at a high level to prevent an activation along this path. Hence, as a generalization of (minimal) cut sets, we define *(minimal) intervention sets *(MISs) in interaction networks as (minimal) sets of elements that are to be removed *or *to be added in order to achieve a certain *intervention task*. By allowing only the removal of elements, the set of MISs coincides with the MCSs.

The computation of the MISs (or the smaller set of MCSs) for a set of activating target paths that involve *negatively *signed arcs is a more difficult task than computing only minimal hitting sets. Indeed, each MIS will still represent a hitting set, because at least one species in each target path must be removed or constitutively provided. The difficulty arises by ambivalent factors which have in some target paths an activating and in others an inhibitory effect upon the end node. We could therefore restrict the interventions to those species that are either pure activators with respect to the target paths (these are allowed to be removed) or pure inhibitors (these are allowed to be added). Using only these species, the MISs could again be computed as the minimal hitting sets.

However, for computing MISs that may also act on ambivalent factors, we present a more general algorithm (here for a given set of *activating *target paths):

(1) In each target path, the involved nodes are labeled by +1 (if the species influences the end node *of the respective path *positively) or by -1 (if the species has a negative influence on the end node *of the respective path*).

(2) Combinations *C*_*i *_of one, two, three, ... distinct removed or activated species are constructed systematically. For each combination *C*_*i*_, it is checked for each target path whether the signal flow from the start node to the end node is interrupted properly. A requirement is that at least one of the positive (+1) species of each path is removed or at least one negative (-1) species is provided (added) by *C*_*i *_(hitting set property). If, for a certain path, *C*_*i *_contains several nodes that are visited by this paths then it is only checked whether the node closest to the end node is attacked properly. When all paths have been attacked (hit) properly by a combination *C*_*i*_, then a new MIS has been found. When constructing further combinations of larger cardinality, the algorithm has to ensure that none of the new combinations contains an earlier found MISs completely.

Of course, this enumerative algorithm is even more time consuming than computing minimal hitting sets and it will become infeasible to compute all MISs in large networks. We may then restrict ourselves to MISs of low cardinality and/or to the subset of MCSs. Besides, the determination of MISs can become even more complicated: it might happen that a MIS attacks all activating target paths correctly but simultaneously destroys an inhibiting path (not contained in the set of target paths) which might then become an activating path. The MCS {I1, I2} of our example represents such a problematic case: it hits the two activating paths to O1 as demanded, but it also attacks the inhibiting path leading from I1 to O1. Thus, the inhibition of E through I1 would be interrupted and it could be sufficient to retain E in an active state enabling the activation of O1. Hence, we would not be sure about the activation status of O1 after removing this cut set. To avoid such side-effects, we may extend our algorithm given above by checking also the consequence of each intervention *C*_*i *_with respect to the non-target paths and exclude combinations that do not fulfill certain criteria.

In a completely analogous fashion, we can also determine MCSs or MISs that repress *inhibitory *paths. For example, removing {I1} is a MCS that attacks the only inhibiting path to O1, alternatively we might use the MISs {#E} or {#C}, where # stands for "constitutively provided". The same issues as discussed above must be taken into account when interrupting a negative path: here, in each target path, only the inhibiting species of the final sink source should be removed whereas the activating nodes can be added. Furthermore, we may also define more complicated intervention tasks, e.g. where some activating and some inhibiting paths are selected as target paths.

#### Jacobian matrix and interaction graph

Several works have highlighted the strong relationships between interaction graphs and the Jacobian matrix **J**, the latter obtained from a dynamical model of the network under investigation [10,35,39]. A dynamic model of a signaling (or any kind of interaction) network is usually described by a system of ordinary differential equations that model the evolution of the *m *network components *x*_1 _... x_*m*_with the time:

x˙=dxdt=(x˙1⋮x˙m)=F(x,t)=(f1(x,t)⋮fm(x,t))     (5)
 MathType@MTEF@5@5@+=feaafiart1ev1aaatCvAUfKttLearuWrP9MDH5MBPbIqV92AaeXatLxBI9gBaebbnrfifHhDYfgasaacH8akY=wiFfYdH8Gipec8Eeeu0xXdbba9frFj0=OqFfea0dXdd9vqai=hGuQ8kuc9pgc9s8qqaq=dirpe0xb9q8qiLsFr0=vr0=vr0dc8meaabaqaciaacaGaaeqabaqabeGadaaakeaaieqacuWF4baEgaGaaiabg2da9maalaaabaGaemizaqMae8hEaGhabaacbiGae4hzaqMae4hDaqhaaiabg2da9maabmaabaqbaeqabmqaaaqaaiqbdIha4zaacaWaaSbaaSqaaiabigdaXaqabaaakeaacqWIUlstaeaacuWG4baEgaGaamaaBaaaleaacqWGTbqBaeqaaaaaaOGaayjkaiaawMcaaiabg2da9Gqadiab9zeagHqaaiab8HcaOiab=Hha4jab8XcaSiab+rha0jab8LcaPiab+1da9maabmaabaqbaeqabmqaaaqaaiab+zgaMnaaBaaaleaacqaFXaqmaeqaaOGaeiikaGIae8hEaGNaeiilaWIaemiDaqNaeiykaKcabaGaeSO7I0eabaGaemOzay2aaSbaaSqaaiabd2gaTbqabaGccqGGOaakcqWF4baEcqGGSaalcqWG0baDcqGGPaqkaaaacaGLOaGaayzkaaGaaCzcaiaaxMaadaqadaqaaiabiwda1aGaayjkaiaawMcaaaaa@5F3E@    

The *m *× *m *Jacobian matrix **J**(**x**) collects the partial derivatives of ***F ***with respect to **x**:

Jik(x)=dfidxk(x)     (6)
 MathType@MTEF@5@5@+=feaafiart1ev1aaatCvAUfKttLearuWrP9MDH5MBPbIqV92AaeXatLxBI9gBaebbnrfifHhDYfgasaacH8akY=wiFfYdH8Gipec8Eeeu0xXdbba9frFj0=OqFfea0dXdd9vqai=hGuQ8kuc9pgc9s8qqaq=dirpe0xb9q8qiLsFr0=vr0=vr0dc8meaabaqaciaacaGaaeqabaqabeGadaaakeaaieqacqWFkbGsdaWgaaWcbaGaemyAaKMaem4AaSgabeaakiabcIcaOiab=Hha4jabcMcaPiabg2da9maalaaabaGaemizaqMaemOzay2aaSbaaSqaaiabdMgaPbqabaaakeaacqWGKbazcqWG4baEdaWgaaWcbaGaem4AaSgabeaaaaGccqGGOaakcqWF4baEcqGGPaqkcaWLjaGaaCzcamaabmaabaGaeGOnaydacaGLOaGaayzkaaaaaa@4480@    

The sign of **J**_*ik*_(**x**) tells whether *x*_*k *_has a (direct) positive or negative influence on *x*_*i *_and sign(**J**(**x**)**) **can thus be seen as the *adjacency *matrix of the underlying interaction graph. In an adjacency matrix **Y**, a non-zero entry for **Y**_*ik *_indicates an edge from node *i *to *k*. Adjacency and incidence matrix are equivalent for describing a graph structure and can be converted into each other: each non-zero element **Y**_*ik *_gets a corresponding column in the incidence matrix.

The sign structure of the Jacobian matrix is, in biological systems, typically constant and reflects, despite its very qualitative nature, fundamental properties of the dynamic system. For example, multistationarity can only occur if a positive circuit exists in the associated interaction graph [39]. Methods for the detection of multistability in a special class of dynamical systems – monotone I/O systems – have been developed by Sontag et al. [36]. Monotone I/O systems possess a monotonicity property that can be checked from the interaction graph spanned by the Jacobian matrix. In fact, having one source species and one sink species, the required monotonicity property is equivalent to our definition of a *total activator *of the sink node. Thus, the methods developed in the previous section may support such studies, where the structure of the Jacobian matrix is analyzed. Having the absolute values of the Jacobian matrix available (which change over time), arcs, paths, and feedback circuits could be assigned an interaction strength useful to identify key elements in the network.

### Boolean networks and (logical) interaction hypergraphs

#### Definitions

**Figure 7:**
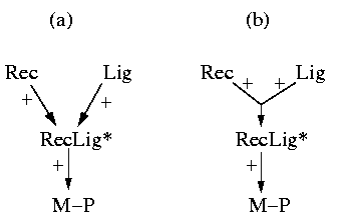
(a) the graphical and (b) the more correct hypergraphical representation of the simple interaction network shown in Figure 1 and 2.

The methods described above consider an interaction as a dependency between two species allowing to employ tools from graph theory. However, in cellular networks, an interaction (edge) often represents a relationship among more than two species (nodes). A typical example is a bimolecular reaction of the form A+B→ C, where three species are involved. The binding of the ligand to the receptor in Figure 2(b) (Rec+Lig→ RecLig*) is such a bimolecular interaction. Using an interaction graph, this reaction is modeled with two arcs (Figure 7(a)), namely Rec→ RecLig* and Lig→ RecLig*, capturing correctly that Rec and Lig have an influence on RecLig*. However, this relaxed representation has shortcomings for a functional interpretation of the network. To exemplify this, consider the minimal cut sets repressing the phosphorylation of M in Figure 7(a). As explained in the previous section, we need to attack all positive paths leading to M-P. There are two positive paths, one starting from Rec and the other from Lig and, thus, {Rec, Lig} would be a minimal cut set. But, intuitively, this cut set is *not *minimal for the real system because both Rec and Lig are required for activating M, and removing only one of the two species is thus sufficient to interrupt the activation of M. (In other words, the existence of a signaling path in an interaction graph does not ensure that a signal can flow along this path.)

This example reveals that a proper consideration of AND-connections between species is required. However, AND-relationships are not possible in graphs but in hypergraphs, which are generalizations of graphs. Similar to a directed graph, a directed hypergraph ***H***=(***V***, ***A***) consists of a set ***V ***of nodes and a set ***A ***of hyperarcs (= directed hyperedges [49]). A hyperarc ***a***connects two subsets of nodes: ***a ***= {***S***,***E***}; ***S***,***E⊂ V***. ***S ***comprises the tail (start) nodes and ***E ***the head (end) nodes of the connection. ***S ***and ***E ***can have arbitrary cardinality, and a graph is a special case of a hypergraph where the cardinality of ***S ***and ***E ***is 1 for all edges.

In our context, without loss of generality, we will usually have only one end node in ***E ***and we interpret a hyperarc as an interaction in which the compound contained in ***E ***is activated by a combined action of the species contained in ***S***. Figure 7(b) depicts the example with the receptor-ligand-complex as a hypergraph in which a hyperarc captures now the AND-connection between Rec and Lig yielding RecLig*.

AND connections facilitate a refined representation of stoichiometric conversions within interaction networks, albeit the precise stoichiometric coefficients are not captured here. Apart from stoichiometric interactions, AND connections allow the description of other dependencies, for example, the case where only the presence of an activator AND the absence of an inhibitor leads to the activation of a certain protein.

In TOYNET, the four nodes (B, C, E, F) have more than one incoming arc (Figure 3). In these nodes it is undetermined how the different stimuli are combined, e.g. whether B AND E are required to activate C or whether one of both is sufficient (B OR E).

We could therefore concatenate all incoming edges in a node by logical operations leading to *Boolean networks *[21,31]. An assumption underlying Boolean networks is to consider only discrete (concentration/activation) levels for each species; in the simplest case a species can only be "off" (= 0 = "inactive" or "absent") and "on" (= 1 = "active" or "present"). Hence, each species is considered as a binary (logical) variable. Next, a Boolean function *f*_*i *_is defined for each node *i *which determines under which conditions *i *is on or off, respectively. *f*_*i *_depends only on those nodes in the interaction graph from which an arc points into species *i*. In general, for constructing a Boolean function, all logical operations like AND, OR, NOT, XOR, NAND can be used. However, here we express each Boolean function by a special representation known as sum of products (SOP; also called (minimal) disjunctive normal form (DNF)) which is possible for any Boolean function [50]. SOP representations require only AND, OR and NOT operators. In a SOP expression, *literals*, which are Boolean variables or negated Boolean variables, are connected by AND's giving *clauses*. Several such AND clauses are then in turn connected by OR's. Using the usual symbols '·' for AND, '+' for OR and '!' for NOT, an example of a SOP expression would be: *f*_*i *_= *x*·*y*·*z *+ *x*·!*z *stating that *f*_*i *_gets value "1" if (*x *AND *y *AND *z *are active) OR (if *x *is active AND *z *is NOT) and "0" else. The SOP expression *f*_*i *_= *x*·*!y *+ !*x*·*y *mimics an XOR gate.

In our context, writing a Boolean function as a SOP has several advantages. First, many biological mechanisms that lead to the activation of a species correspond directly to SOP representations. Second, by using SOPs, the structure of a Boolean network can be represented and depicted intuitively as a hypergraph: each hyperarc pointing into a node *i *is an AND clause of other nodes and represents one way of activating *i; *hence, all hyperarcs ending in *i *are OR'ed together. A hyperarc carries a signal flow to its end node and the binary value of the flow depends on the state of all its start nodes. In the following, such a hypergraph induced by a minimal SOP representation of a Boolean network will be called a *logical interaction hypergraph (LIH*).

**Figure 8:**
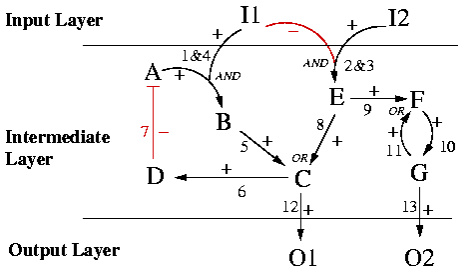
Logical interaction hypergraph of TOYNET (compare with interaction graph in Figure 3).

In Figure 8 a possible instance of a LIH compatible with the interaction graph of TOYNET in Figure 3 is depicted. In each of the four nodes with more than one incoming arc, the logical concatenation has now been specified. For example, B is now activated if A AND I1 are active simultaneously (hyperarc "1&4"). In contrast, C is activated if B OR E is present (active), and F is active if E OR G are in an active state. Hence, C and F retain their graph-like structure.

Inhibiting arcs in the interaction graph are interpreted in the corresponding LIH as NOT-operations. Thus, arc 7 is now interpreted as "A is active if D is not present". Since arc 2 and 3 in Figure 3 have been combined with an AND in Figure 8, we interpret this new hyperarc as "E becomes activated if I2 is present AND I1 NOT". Hence, in contrast to inhibiting arcs in interaction graphs, in general we do not assign a minus sign (a NOT) to the complete hyperarc, but to its negative branches (see hyperarc 2&3 in Figure 8), whereas all other branches get positive signs. Due to the assignment of signs LIHs can formally be seen as *signed directed hypergraphs*.

The pure logical description of a signaling or regulatory network works well when the activation (inhibition) of a species by others follows a sigmoid curve [21]. Problems that might arise while describing a real network within the logical framework and possible solutions are discussed in a later section.

LIHs can be formally represented and stored in a similar way as interaction graphs. The underlying hypergraph is stored by an *m *× *n *incidence matrix **B **in which the rows correspond to the species and the columns to the *n *hyperarcs. If species *i *is contained in the set of start (tail) nodes of a hyperarc *k *then **B**_*ik *_= -1, if *i *is the endpoint (head) of hyperarc *k *then **B**_*ik *_= 1, and if *i *is not involved in *k *we have **B**_*ik *_= 0. For storing the NOTs operating on certain species in a hyperarc we may use another *m *× *n *matrix **U **that stores in **U**_*ik *_a "1" if species *i *enters the hyperarc *k *with its negated value and "0" else. Accordingly, the incidence matrix **B **for the LIH of TOYNET (Figure 8) reads

B=1&42&35678910111213(−1−1(*)0000000000−1000000000−1000100000010−100000000001−101000−100001−1(*)00000001000−1−100000000001−110000000001−10−10000000001000000000001)I1I2ABCDEFGO1O2     (7)
 MathType@MTEF@5@5@+=feaafiart1ev1aaatCvAUfKttLearuWrP9MDH5MBPbIqV92AaeXatLxBI9gBaebbnrfifHhDYfgasaacH8akY=wiFfYdH8Gipec8Eeeu0xXdbba9frFj0=OqFfea0dXdd9vqai=hGuQ8kuc9pgc9s8qqaq=dirpe0xb9q8qiLsFr0=vr0=vr0dc8meaabaqaciaacaGaaeqabaqabeGadaaakeaaieqacqWFcbGqcqGH9aqpfaqabeGacaaabaqbaeqabeWcaaaaaeaacWaGaIx=aaaIXaqmcWaGaIx=aaGGMaGjcWaGaIx=aaaI0aanaeaacWaGacq=aaaIYaGmcWaGacq=aaGGMaGjcWaGacq=aaaIZaWmaeaacWaGaYt=aaaI1aqnaeaacWaGacF=aaaI2aGnaeaacWaGac3=aaaI3aWnaeaacWaGacmaaaaI4aaoaeaacWaGaIwaaaaI5aqoaeaacWaGaICaaaaIXaqmcWaGaICaaaaIWaamaeaacWaGacEaaaaIXaqmcWaGacEaaaaIXaqmaeaacWaGaIFaaaaIXaqmcWaGaIFaaaaIYaGmaeaacWaGaYHaaaaIXaqmcWaGaYHaaaaIZaWmaaaabaaabaWaaeWaaeaafaqabeWclaaaaaaaaeaacqGHsislieaacqGFXaqmaeaacqGHsislcqGFXaqmcqGFOaakcqGFQaGkcqGFPaqkaeaacqGFWaamaeaacqGFWaamaeaacqGFWaamaeaacqGFWaamaeaacqGFWaamaeaacqGFWaamaeaacqGFWaamaeaacqGFWaamaeaacqGFWaamaeaacqGFWaamaeaacqGHsislcqGFXaqmaeaacqGFWaamaeaacqGFWaamaeaacqGFWaamaeaacqGFWaamaeaacqGFWaamaeaacqGFWaamaeaacqGFWaamaeaacqGFWaamaeaacqGFWaamaeaacqGHsislcqGFXaqmaeaacqaIWaamaeaacqGFWaamaeaacqaIWaamaeaacqGFXaqmaeaacqGFWaamaeaacqaIWaamaeaacqGFWaamaeaacqGFWaamaeaacqGFWaamaeaacqGFWaamaeaacqaIXaqmaeaacqGFWaamaeaacqGHsislcqGFXaqmaeaacqGFWaamaeaacqGFWaamaeaacqGFWaamaeaacqGFWaamaeaacqGFWaamaeaacqGFWaamaeaacqGFWaamaeaacqGFWaamaeaacqGFWaamaeaacqGFWaamaeaacqGFXaqmaeaacqGHsislcqaIXaqmaeaacqGFWaamaeaacqGFXaqmaeaacqGFWaamaeaacqaIWaamaeaacqGFWaamaeaacqGHsislcqaIXaqmaeaacqGFWaamaeaacqGFWaamaeaacqGFWaamaeaacqGFWaamaeaacqGFXaqmaeaacqGHsislcqaIXaqmcqGGOaakcqGGQaGkcqGGPaqkaeaacqGFWaamaeaacqGFWaamaeaacqGFWaamaeaacqGFWaamaeaacqGFWaamaeaacqGFWaamaeaacqGFWaamaeaacqGFXaqmaeaacqGFWaamaeaacqGFWaamaeaacqGFWaamaeaacqGHsislcqaIXaqmaeaacqGHsislcqaIXaqmaeaacqGFWaamaeaacqGFWaamaeaacqGFWaamaeaacqGFWaamaeaacqGFWaamaeaacqGFWaamaeaacqGFWaamaeaacqGFWaamaeaacqGFWaamaeaacqGFWaamaeaacqGFXaqmaeaacqGHsislcqaIXaqmaeaacqGFXaqmaeaacqaIWaamaeaacqGFWaamaeaacqGFWaamaeaacqGFWaamaeaacqGFWaamaeaacqGFWaamaeaacqGFWaamaeaacqGFWaamaeaacqGFWaamaeaacqGFXaqmaeaacqGHsislcqaIXaqmaeaacqGFWaamaeaacqGHsislcqGFXaqmaeaacqGFWaamaeaacqGFWaamaeaacqGFWaamaeaacqGFWaamaeaacqGFWaamaeaacqGFWaamaeaacqGFWaamaeaacqGFWaamaeaacqGFWaamaeaacqGFXaqmaeaacqGFWaamaeaacqGFWaamaeaacqGFWaamaeaacqGFWaamaeaacqGFWaamaeaacqGFWaamaeaacqGFWaamaeaacqGFWaamaeaacqGFWaamaeaacqGFWaamaeaacqGFWaamaeaacqGFXaqmaaaacaGLOaGaayzkaaaabaqbaeqabSqaaaaaaeaacqGFjbqscqGFXaqmaeaacqGFjbqscqGFYaGmaeaacqGFbbqqaeaacqGFcbGqaeaacqGFdbWqaeaacqGFebaraeaacqGFfbqraeaacqGFgbGraeaacqGFhbWraeaacqGFpbWtcqGFXaqmaeaacqGFpbWtcqGFYaGmaaaaaiaaxMaacaWLjaGaeiikaGIaeG4naCJaeiykaKcaaa@FA2F@    

To be concise, the two non-zeros entries of **U **are indicated by an asterisk in the incidence matrix.

Representing a Boolean network as a LIH we can easily reconstruct the underlying interaction graph from the matrices **B **and **U: **we simply split up the hyperarcs having more than one start node (or/and more than one end node in the general case). Thus, a hyperarc with *d *start and *g *end nodes is converted into *d·g *arcs in the interaction graph. The sign of each arc in the graph model can be obtained from **U**. The reverse, the reconstruction of the LIH from the interaction graph, is not possible in a unique manner underlining the non-deterministic nature of interaction graphs.

#### Time in Boolean networks

A *logical interaction hypergraph *describes only the static structure of a Boolean network. However, it is the *dynamic *behavior of Boolean networks that has been analyzed intensely in the context of biological (especially genetic) systems [21,31,51]. For studying the evolution of a logical system we need to introduce the (discrete) time variable *t *and a state vector **x**(*t*) that captures the logical values of the *m *species at time point *t*. Two fundamental strategies exist to derive the new state vector **x**(*t+1) *from the current state **x**(*t*). In the *synchronous *model, the logical value of each node *i *is updated by evaluating its Boolean function *f*_*i *_with the current state vector: *x*_*i *_(*t+1*) = *f*_*i*_(**x**(*t*)). Synchronous models are deterministic but assume for all interactions the same time delay which is often too unrealistic for biological systems [21]. In the *asynchronous *model, we select any (but only one) node *i *whose current state is unequal to its associated Boolean function: *x*_*i *_(*t*) ≠ *f*_*i*_(**x**(*t*)). Only this node switches in the next iteration. Since there are, in general, degrees of freedom in choosing the switching node, this description is non-deterministic. The advantage is that the complete spectrum of potential trajectories is captured, albeit the graph of sequences is usually very dense, complicating its analysis in large systems. The asynchronous description becomes (more) deterministic if time delays for activation and inhibition events are known [21].

We are now approaching the main part of this section.

#### Logical steady-state analysis

An important characteristic of the dynamic behavior of Boolean networks, which is equivalent for both asynchronous and synchronous descriptions, is the set of *logical steady states (LSSs)*. LSSs are state vectors **x**^s ^obeying xis
 MathType@MTEF@5@5@+=feaafiart1ev1aaatCvAUfKttLearuWrP9MDH5MBPbIqV92AaeXatLxBI9gBaebbnrfifHhDYfgasaacH8akY=wiFfYdH8Gipec8Eeeu0xXdbba9frFj0=OqFfea0dXdd9vqai=hGuQ8kuc9pgc9s8qqaq=dirpe0xb9q8qiLsFr0=vr0=vr0dc8meaabaqaciaacaGaaeqabaqabeGadaaakeaacqWG4baEdaqhaaWcbaGaemyAaKgabaGaem4Camhaaaaa@311C@ = *f_i_*(**x**^s^) for all nodes *i*. Hence, in LSS, the state of each node is consistent with the value of its associated Boolean function and, therefore, once a Boolean network has moved into a logical steady state, it will stop to switch and then retain this state.

In the following, we will focus on logical steady state analysis (thus circumventing any interpretation problems that might arise by choosing synchronous or asynchronous description), which suffices for a number of applications, especially for predicting potential functional states in signaling or regulatory networks.

Given a Boolean network we may enumerate all possible LSSs [52]. However, this is computationally difficult in large networks. Besides, we are often interested in particular LSSs that can be reached from a given initial state **x**^0^. In some cases, we only know a fraction of all initial node values. For example, a typical scenario in signaling networks would be that initial values from species in the input layer are known (specifying which external signals reach the cell and which not), and we would like to know how the (logical) integration and propagation of these input signals generate a certain logical pattern in the output layer. Of course, we have to "wait" until the signals reach the bottom of the network and, for obtaining a unique answer, there should be a time point from which the states will not change in the future. This is equivalent to determining the LSS in which the network will run from a given starting point.

**Figure 9:**
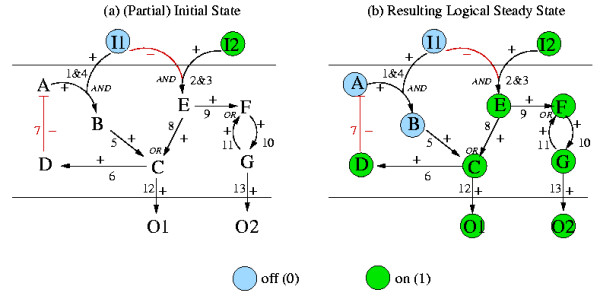
Example of a logical steady state in TOYNET resulting from a particular set of initial states in the input layer.

In a possible scenario for TOYNET, the initial values of the source species I1 and I2 might be known to be xI10
 MathType@MTEF@5@5@+=feaafiart1ev1aaatCvAUfKttLearuWrP9MDH5MBPbIqV92AaeXatLxBI9gBaebbnrfifHhDYfgasaacH8akY=wiFfYdH8Gipec8Eeeu0xXdbba9frFj0=OqFfea0dXdd9vqai=hGuQ8kuc9pgc9s8qqaq=dirpe0xb9q8qiLsFr0=vr0=vr0dc8meaabaqaciaacaGaaeqabaqabeGadaaakeaacqWG4baEdaqhaaWcbaacbiGae8xsaKKaeGymaedabaGaeGimaadaaaaa@3152@ = 0 and xI20
 MathType@MTEF@5@5@+=feaafiart1ev1aaatCvAUfKttLearuWrP9MDH5MBPbIqV92AaeXatLxBI9gBaebbnrfifHhDYfgasaacH8akY=wiFfYdH8Gipec8Eeeu0xXdbba9frFj0=OqFfea0dXdd9vqai=hGuQ8kuc9pgc9s8qqaq=dirpe0xb9q8qiLsFr0=vr0=vr0dc8meaabaqaciaacaGaaeqabaqabeGadaaakeaacqWG4baEdaqhaaWcbaacbiGae8xsaKecbaGae4NmaidabaGaeGimaadaaaaa@3153@ = 1, whereas the initial states of all other nodes are unknown (Figure 9(a)). The states of I1 and I2 will not change anymore because I1 and I2 have no predecessor in the hypergraph model. Assuming that each interaction has a finite time delay, E must become active and B inactive. From these fixed values we can conclude that C and F will definitely become active (by E) at a certain time point and not change this state in the future. Proceeding further in the same way, we can resolve the complete LSS resulting from the given initial values of I1 and I2 (Figure 9(b)).

The last example illustrated that partial knowledge on initial values, especially from the source nodes, can be sufficient to determine the resulting LSS uniquely. However, in general, several LSSs might result from a given set of initial values or a LSS may not exist at all. For example, if we only know xI20
 MathType@MTEF@5@5@+=feaafiart1ev1aaatCvAUfKttLearuWrP9MDH5MBPbIqV92AaeXatLxBI9gBaebbnrfifHhDYfgasaacH8akY=wiFfYdH8Gipec8Eeeu0xXdbba9frFj0=OqFfea0dXdd9vqai=hGuQ8kuc9pgc9s8qqaq=dirpe0xb9q8qiLsFr0=vr0=vr0dc8meaabaqaciaacaGaaeqabaqabeGadaaakeaacqWG4baEdaqhaaWcbaacbiGae8xsaKecbaGae4NmaidabaGaeGimaadaaaaa@3153@ = 1 in TOYNET nothing can be concluded regarding a LSS (except that I2 will retain its state). If no complete LSS can be concluded uniquely from initial values, there might nevertheless be a subset of nodes that will reach a state in which they will remain for the future. For example, setting xI10
 MathType@MTEF@5@5@+=feaafiart1ev1aaatCvAUfKttLearuWrP9MDH5MBPbIqV92AaeXatLxBI9gBaebbnrfifHhDYfgasaacH8akY=wiFfYdH8Gipec8Eeeu0xXdbba9frFj0=OqFfea0dXdd9vqai=hGuQ8kuc9pgc9s8qqaq=dirpe0xb9q8qiLsFr0=vr0=vr0dc8meaabaqaciaacaGaaeqabaqabeGadaaakeaacqWG4baEdaqhaaWcbaacbiGae8xsaKKaeGymaedabaGaeGimaadaaaaa@3152@ = 1 E will definitely become inactivated after some time (again, finite time delay is assumed). Since in this scenario nothing further can be derived for other nodes, we would say that *x_I1 _*= 1 and *x_E _*= 0 are *partial LSSs *for the initial value set {xI10
 MathType@MTEF@5@5@+=feaafiart1ev1aaatCvAUfKttLearuWrP9MDH5MBPbIqV92AaeXatLxBI9gBaebbnrfifHhDYfgasaacH8akY=wiFfYdH8Gipec8Eeeu0xXdbba9frFj0=OqFfea0dXdd9vqai=hGuQ8kuc9pgc9s8qqaq=dirpe0xb9q8qiLsFr0=vr0=vr0dc8meaabaqaciaacaGaaeqabaqabeGadaaakeaacqWG4baEdaqhaaWcbaacbiGae8xsaKKaeGymaedabaGaeGimaadaaaaa@3152@ = 1}. Note that these two partial steady states would not change when we specified more or even all initial values.

We have conceived an algorithm which derives partial LSSs that follow from a given set of initial values (if for each node a partial LSS can be found, then a unique and complete LSS exists for the set of initial values). The iterative algorithm uses the following rules in the logical hypergraph model:

• initial values of source nodes will not change in the future, hence, are partial LSSs

• if species *i *has a proved partial LSS of 0, all hyperarcs in which *i *is involved with its non-negated value have a zero flow

• if species *i *has a proved partial LSS of 1, all hyperarcs in which *i *is involved with its negated value have a zero flow

• if all hyperarcs pointing into node *i *have a zero flow, then *i *has a partial LSS of 0

• if all start nodes of a hyperarc have a partial LSS of 1 (or of 0 for those start nodes entering the hyperarc with the negated value) then a partial LSS of 1 follows for the end node of this hyperarc

• knowing all the positive feedback circuits in the system, we can check whether there is a "self-sustaining" positive circuit where the known initial state values of the involved nodes guarantee a partial LSS for all the nodes in this cycle (see comments below)

In each loop, the algorithm tries to identify new partial LSSs (following from the current set of partial LSSs already identified) until no further ones can be found. Setting initial values in the input layer, this can be envisioned as a propagation of signals through the interaction network until signals reach nodes where the available information is not sufficient to derive a unique LSSs.

Generally, in logical interaction hypergraphs where the underlying interaction graph has no feedback loop (i.e. is acyclic), specification of the initial values of all the source nodes will always result in a unique and complete LSS since the signals can be propagated step by step from top-down to the output layer. In general, if all initial values are known for the input layer, non-uniqueness or even non-existence of partial LSSs can only be generated by feedback loops. The partial LSSs of nodes involved in positive feedbacks do often depend on the initial values of all the nodes in this loop. For example, defining xI20
 MathType@MTEF@5@5@+=feaafiart1ev1aaatCvAUfKttLearuWrP9MDH5MBPbIqV92AaeXatLxBI9gBaebbnrfifHhDYfgasaacH8akY=wiFfYdH8Gipec8Eeeu0xXdbba9frFj0=OqFfea0dXdd9vqai=hGuQ8kuc9pgc9s8qqaq=dirpe0xb9q8qiLsFr0=vr0=vr0dc8meaabaqaciaacaGaaeqabaqabeGadaaakeaacqWG4baEdaqhaaWcbaacbiGae8xsaKecbaGae4NmaidabaGaeGimaadaaaaa@3153@ = 0 we can conclude a partial LSSs of zero for E in TOYNET (Figure 8), but, among others, the values of F, G and O2 remain unknown although the only connection to a source node leads via E. The reason is that F and G build up a positive feedback loop which cannot be resolved without knowledge on further initial values. If we know, additionally to xI20
 MathType@MTEF@5@5@+=feaafiart1ev1aaatCvAUfKttLearuWrP9MDH5MBPbIqV92AaeXatLxBI9gBaebbnrfifHhDYfgasaacH8akY=wiFfYdH8Gipec8Eeeu0xXdbba9frFj0=OqFfea0dXdd9vqai=hGuQ8kuc9pgc9s8qqaq=dirpe0xb9q8qiLsFr0=vr0=vr0dc8meaabaqaciaacaGaaeqabaqabeGadaaakeaacqWG4baEdaqhaaWcbaacbiGae8xsaKecbaGae4NmaidabaGaeGimaadaaaaa@3153@ = 0, that xF0=xG0
 MathType@MTEF@5@5@+=feaafiart1ev1aaatCvAUfKttLearuWrP9MDH5MBPbIqV92AaeXatLxBI9gBaebbnrfifHhDYfgasaacH8akY=wiFfYdH8Gipec8Eeeu0xXdbba9frFj0=OqFfea0dXdd9vqai=hGuQ8kuc9pgc9s8qqaq=dirpe0xb9q8qiLsFr0=vr0=vr0dc8meaabaqaciaacaGaaeqabaqabeGadaaakeaacqWG4baEdaqhaaWcbaGaemOrayeabaGaeGimaadaaOGaeyypa0JaemiEaG3aa0baaSqaaiabdEeahbqaaiabicdaWaaaaaa@3510@ = 1 then F and G will always keep each other activated so that we can infer a partial LSS of 1 for F, G and O2 (this is the last rule in the list given above). If we have instead xF0=xG0
 MathType@MTEF@5@5@+=feaafiart1ev1aaatCvAUfKttLearuWrP9MDH5MBPbIqV92AaeXatLxBI9gBaebbnrfifHhDYfgasaacH8akY=wiFfYdH8Gipec8Eeeu0xXdbba9frFj0=OqFfea0dXdd9vqai=hGuQ8kuc9pgc9s8qqaq=dirpe0xb9q8qiLsFr0=vr0=vr0dc8meaabaqaciaacaGaaeqabaqabeGadaaakeaacqWG4baEdaqhaaWcbaGaemOrayeabaGaeGimaadaaOGaeyypa0JaemiEaG3aa0baaSqaaiabdEeahbqaaiabicdaWaaaaaa@3510@ = 0, we derive a 0 for the partial LSS of these three nodes. If one of the two nodes F and G has an initial value of 1 and the other 0, nothing can be derived since the positive loop might become fully activated or fully deactivated. However, what can be confirmed in these simple examples is that positive feedback loops induce multistationarity. It is noteworthy that continuous dynamic models of networks with positive feedbacks will depend, apart from kinetic parameters, in a similar fashion on initial state values.

In contrast, negative feedback loops are not sensitive against initial values but they can be the source of oscillations, preventing hence the existence of LSSs. In TOYNET we have one negative feedback loop which can potentially generate oscillations, for example, when we set xI10
 MathType@MTEF@5@5@+=feaafiart1ev1aaatCvAUfKttLearuWrP9MDH5MBPbIqV92AaeXatLxBI9gBaebbnrfifHhDYfgasaacH8akY=wiFfYdH8Gipec8Eeeu0xXdbba9frFj0=OqFfea0dXdd9vqai=hGuQ8kuc9pgc9s8qqaq=dirpe0xb9q8qiLsFr0=vr0=vr0dc8meaabaqaciaacaGaaeqabaqabeGadaaakeaacqWG4baEdaqhaaWcbaacbiGae8xsaKKaeGymaedabaGaeGimaadaaaaa@3152@ = 1. Then, C cannot be activated via E. Assuming an initial value of 0 for C (the same conclusion would be drawn with 1), D becomes deactivated and thus A actived. Due to the partial LSS of 1 for I1 we get an activation of B and then of C and D which in turn inhibits A leading in the next round to a deactivation of B, C and D and so on. The logical states within this circuit and downstream of it (O1) will thus never reach a steady state. As shown in [21], oscillatory behavior in logical models corresponds to oscillations or a stable equilibrium (lying somehow between the fully activated and fully inactivated level) in the associated continuous model, depending on the chosen parameters. Negative feedback loops can thus impede predictions on the basis of logical steady states, but they also point to network structures whose parametrization will have great impact on the dynamic behavior.

Note that feedback loops do not always prevent predictions on (partial) LSSs as can be seen by the example in Figure 9, it depends on the given initial values.

Such a logical steady state or "signal flow" analysis (SFA) as presented herein shares similarities with the established method of metabolic flux analysis [53]. In MFA, uptake and excretion rates of cells are measured in order to reconstruct the intracellular flux distribution within a metabolic network. MFA relies on the quasi-steady state assumption, similarly as SFA relies on LSS. However, whereas MFA tries to reconstruct the reaction rates along the edges and nothing can be said on the states of the species, the goal of SFA is to determine the steady states of the nodes (belonging to a given activation scheme) from which then the signal flows along the edges follow. It is noteworthy that the calculability of unknown reaction rates in MFA depends only on the *set *of known rates [54], whereas in SFA the set of given initial states *and their respective values *determine the unique calculability of (partial) LSSs.

#### Applications of logical steady state analysis

The LSS analysis introduced herein offers a number of applications for studying functional aspects in cellular interaction networks:

##### Input-output behavior

Imposing different patterns of signals in the input layer one may check which species become activated or inhibited in the intermediate and, in particular, in the output layer. This can also be simulated in combination with different initial state values for certain intermediate nodes, albeit this will have an influence on the LSS only in connection with positive feedbacks, as shown above.

##### Mutants and interventions

The changes in signals flows and in the input-output behavior occurring in a manipulated or malfunctioning network can be studied by removing or adding elements or by fixing the states of certain species in the network. In TOYNET, for example, if we want to study the effect of a mutant missing F (or the effect of adding an inhibitor for F) we may remove species F from the network (or, equivalently, fix the state of F to zero) and compute then the partial LSSs again. We will see that, independently of a given pattern in the input layer, G and O1 will be assigned a partial LSS of 0. Removing elements often changes not only the *values*, but also the *determinacy *of partial LSSs.

##### Minimal cut sets (MCSs) and minimal intervention sets (MISs)

The definiton of MCSs and MISs in logical interaction hypergraphs is similar as in interaction graphs: a MCS is a minimal (irreducible) set of species whose removal will prevent a certain response or functionality as defined by an intervention goal. In the more general MISs we permit, additionally to cuts, also the constitutive *activation *of certain compounds. Two examples in TOYNET: removing F is a MCS for repressing an activation of G and O2. Assuming an initial state of zero for the species in the intermediate layer, adding I1 and removing B would be a proper MIS for repressing the activation of O1 and O2. Note that in the interaction graph of TOYNET, this intervention would not suffice to attack all activating paths leading from the input layer to O1 and O2 (path P4 not attacked, Figure 5). This example underscores again that MCSs and MISs in interaction hypergraphs are usually smaller than those obtained from the underlying interaction graph, simply because more constraints are added by logical combinations. However, the determination of MCSs, and let alone MISs, in logical interaction hypergraphs is combinatorially complicated as in interaction graphs, in particular when negative signs (NOTs) occur. Here, we can only propose a "brute-force" approach where the LSS analysis serves algorithmically as an oracle: we check systematically for each combination of one, two, three ... knocked out (for MISs also of permanently activated) nodes in the network how this affects the (partial) LSSs, possibly in combination with a given scenario of initial states. From the resulting partial LSSs we can decide whether our intervention goal has been achieved or not. To compute only *minimal *cut or intervention sets, further combinations with a cut or intervention set already satisfying our intervention goal have to be avoided. The algorithm can be stopped when a user-given maximum cardinality for the MCSs/MISs has been reached.

##### Backward propagation

The methods described above compute partial LSSs actually only by forward propagation of signals, but one may also do the opposite, e.g. fixing values in the output layer and tracing back the required states of nodes in the intermediate and input layer using similar rules as for forward propagation.

##### Network expansion methods

There is an interesting relationship between our LSS analysis and network expansion methods proposed by Ebenhöh et al. [55]. Network expansion allows for checking which metabolites can in principle be produced from a provided set of start species within a metabolic (stoichiometric) reaction network. This is a special case in our logical framework. Briefly, metabolic networks are per se hypergraphs and can thus be represented as a LIH by using only AND's (each reaction is an AND clause of its reactants; stoichiometric coefficients are not considered) and OR's. Hence, no inhibiting interactions exist. We may then put the supplied set of available species in the input layer, set the initial values of all other species to zero and compute then the LSS. Note that, according to the explanations given above, a complete LSS will always be found since all initial values are given and no negative feedback circuit exists. Therefore, the computed LSS indicates which species can be produced from the input set and which not.

#### Extensions for the logical description of interaction networks

Several extensions and refinements of the logical framework can be introduced which allow a more appropriate description of real signaling and regulatory networks:

(1) As already proposed and applied by Thomas et al. [21], the discretization in more than two levels is in principle possible. This mimics the fact, that in reality multiple relevant threshold values for a species may exist. A refined discretization could be relevant, for instance, for a species that activates/inhibits more than one species (with different threshold levels). Another relevant situation occurs if a species can be activated via two paths (connected by an OR; see species C in TOYNET): the activation via *both *paths might be significantly stronger than by only *one*. However, considering several activation levels for a certain species forces one to often consider multiple levels for elements downstream or/and upstream of this species, increasing hence the complexity of the network, and requiring detailed knowledge which is often not available.

(2) As we have seen, negative feedback can limit the predictability in LSS analysis. However, in cellular networks, negative feedbacks become activated often upon a certain time period after an activation event occurs, for example, when gene expression is involved. This might be considered by classifying species and/or hyperedges by assigning a discrete *time constant *(or time scale) τ to each element telling us whether this network element appears in an early (τ = 1) or late (τ = 2) state. Using the sub-network with all elements having a time constant of τ = 1 for the first simulation and then using the computed LSSs as initial values for computing the second round (where the complete network is considered) leads often to more realistic results. As in the case of multiple levels, this extension requires a more detailed knowledge about the network under consideration. An example in TOYNET (Figure 8): we may assume that D is a factor that is transcriptionally regulated by C, thus, arc 6 has a time constant of τ = 2 and all others have τ = 1. Setting the initial values I1 = 1, I2 = 0 and D = 0 and computing the LSSs for τ = 1 activation of C and O1 occurs. We can then fix the state of D (D = 1) and get then a complete deactivation of C and O1.

(3) In real signaling and regulatory networks, it is sometimes difficult to decide whether arcs from the interaction graph have to be linked by an AND or an OR in the interaction hypergraph. For example, in TOYNET, species E is inhibited by factor I1 and activated by factor I2. If I1 has a very strong inhibiting effect on E we may formulate the hyperarc as done in Figure 8, suggesting that I1 must not be active for activating E. However, if the interaction strength of both I1 and I2 with respect to E is at the same level (i.e. additive) neither "NOT(I1) OR I2" nor "NOT(I1) AND I2" would reflect the real situation. Indeed, this is a recurring situation in signaling networks, where often a balance between different signals determines the activation of a certain element. At this point it could be helpful to use logical operations that have a partially incomplete truth table. In the latter example we could say that E is active if (NOT(I1) AND I2) and E is inactive if (I1 AND NOT(I2)). For the other two possible cases, no decision could be made along this hyperedge. Of course, modeling uncertainty in this way will limit the determinacy but on the other hand a determined result with this model allows a safer interpretation.

### Analyzing interaction networks using *CellNetAnalyzer*

**Figure 10:**
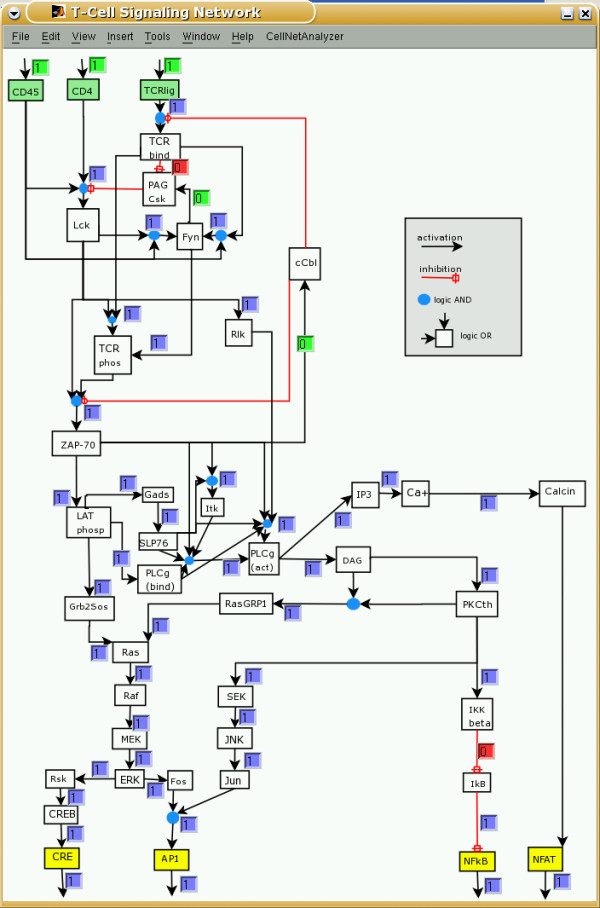
Screenshot of the *CellNetAnalyzer *model for T-cell activation. Each arrow finishing on a species box represents a hyperarc and all the hyperarcs pointing into a species box are OR connected. In the shown "early-event" scenario, the feedbacks were switched off whereas all input arcs are active. The resulting logical steady state was then computed. Text boxes display the signal flows along the hyperarcs (green boxes: fixed values prior computation; blue boxes: hyperarcs activating a species (signal flow is 1); red boxes: hyperarcs which are not active (signal flow is 0)).

We have integrated many of the methods and algorithms described herein in our software tool *CellNetAnalyzer*, which is a MATLAB package and the successor of *FluxAnalyzer *[56]. Whereas *FluxAnalyzer *was originally developed for structural and functional analysis of metabolic networks, *CellNetAnalyzer *extends these capabilities consequently to the structural analysis of signaling and regulatory networks. Apart from stoichiometric (metabolic) reaction networks, *CellNetAnalyzer *supports now also the composition of logical interaction hypergraphs using AND, OR and NOT connections. Whenever needed, the underlying interaction graph can be deduced from the interaction hypergraph. Alternatively, by using only OR's and NOT's, arbitrary interaction graphs can be constructed. As in *FluxAnalyzer*, the network model can be linked with externally created graphics visualizing the network. User interfaces (text boxes) enable data input and output directly in these interactive maps (see screenshot in Figure 10). New functions for graph-theoretical and logical analysis have been integrated into the user menu; the results from computations are directly displayed within the interaction maps or in separate windows. The functions include:

• large-scale computation of all (positive and negative) signaling paths connecting inputs with outputs or of all signaling paths between a given pair of nodes; statistical analysis of these paths

• large-scale computation of all (positive and negative) feedback loops; statistical analysis of these routes

• computation of minimal cut sets for a given set of paths or/and loops

• computation of distance (shortest paths) matrices – separately for positive and negative paths

• large-scale dependency analysis: identification of (total) activators, (total) inhibitors and ambivalent factors for a given species; display of the dependency matrix

• computation of (partial) logical steady states from a given set of initial state values

• computation of (logical) minimal cut sets repressing or provoking a user-defined behavior in the logical network

To illustrate the ability of our approach to deal with real complex signaling networks, we have set-up and analyzed in *CellNetAnalyzer *a logical model of T-cell activation (Figure 10), which will be discussed in the next section.

*CellNetAnalyzer *is free for academic purposes (see web-site [57]).

### Logical model of T-cell activation

#### T-cell activation and the molecular mechanisms behind

T-lymphocytes play a key role within the immune system: Cytotoxic, CD8^+^, T-cells destroy cells infected by viruses or malignant cells, and CD4^+ ^helper T-cells coordinate the functions of other cells of the immune system, such as B-lymphocytes and monocytes [58]. Loss or dysfunction, especially of CD4^+ ^T-cells (as it occurs e.g. in the course of HIV infection or in immuno-deficiencies) has severe consequences for the organism and results in susceptibility to viral and fungal infections as well as in the development of malignancies. The importance of T-cells for immune homeostasis is due to their ability to specifically recognize foreign, potentially dangerous, agents and, subsequently, to initiate a specific immune response that is aimed at eliminating them. T-cells detect foreign antigens by means of their T-Cell Receptor (TCR) which recognizes peptides only when presented on MHC (Major Histocompatibility Complex) molecules. The peptides that are recognized by the TCR are typically derived from foreign (e.g. bacterial, viral) proteins and are generated by proteolytic cleavage within so called antigen presenting cells (APCs). Subsequent to their production the peptides are loaded onto the MHC-molecules and the assembled peptide/MHC-complex is then transported to the cell surface of the APC were it can be recognized by T-cells. The whole process of antigen uptake, proteolytic cleavage, peptide loading onto MHC, transport of the peptide/MHC complex to the surface of the APC and the recognition of the peptide/MHC-complex by the TCR is called antigen presentation and provides the molecular basis for the fine specificity of the adaptive immune response.

The binding of peptide/MHC to the TCR, and the additional binding of a different region of the MHC molecules to so called co-receptors (CD4 in the case of helper T-cells and CD8 in the case of cytotoxic T-cells), initiates a plethora of signaling cascades within the T-cell. As a result, several transcription factors – most importantly, AP1, NFAT and NFκB – are activated. These transcription factors, in turn, control the cell's fate, e.g. whether it becomes activated and proliferates [59] or not.

In the following, a logical model describing some of the main steps involved in the activation of CD4^+ ^helper T-cells (also applicable for CD8^+ ^cytotoxic T-cells) will be briefly introduced and analyzed (see Figure 10 and Table 2). Several players, in particular, some whose role and activation is not completely understood, are not included in our model and thus their effects are not considered or lumped with others. Additionally, in several, currently still controversial cases, we have assumed one of the possible hypotheses; however, this does not mean that we propose this to be the correct description of the TCR-induced signaling network; we just want to demonstrate the applicability of our approach on a realistic, complex case. It is out of the scope of this paper to analyze the complete, highly-complex signaling machinery of a T-cell.

**Table 2:** The hyperarcs of the logical T-cell signaling model (see Figure 10). Exclamation mark ('!') denotes a logical NOT and dots within the equations indicate AND operations.

→ CD45
→ CD8
→ TCRlig
AP1 →
Ca → Calcin
Calcin → NFAT
CRE →
CREB → CRE
DAG → PKCth
ERK → Fos
ERK → Rsk
Fyn → PAGCsk
Fyn → TCRphos
Gads → SLP76
Grb2Sos → Ras
!IkB → NFkB
!IKKbeta → IkB
IP3 → Ca
JNK → Jun
Jun·Fos → AP1
LAT → Gads
LAT → Grb2Sos
LAT → PLCgbind
Lck·CD45 → Fyn
Lck → Rlk
MEK → ERK
NFAT →
NFkB →
!PAGCsk·CD8·CD45 → Lck
PKCth·DAG → RasGRP1
PKCth → IKKbeta
PKCth → SEK
PLCg(act) → DAG
PLCg(act) → IP3
Raf → MEK
Ras → Raf
RasGRP1 → Ras
Rsk → CREB
SEK → JNK
TCRbind·CD45 → Fyn
TCRbind·Lck → TCRphos
!TCRbind → PAGCsk
TCRlig·!cCbl → TCRbind
TCRphos·Lck·!cCbl → ZAP70
ZAP70·SLP76·PLCg(bind)·Itk → PLCg(act)
ZAP70·SLP76 → Itk
ZAP70 → cCbl
ZAP70 → LAT
ZAP70·SLP76·Rlk·PLCg(bind) → PLCg(act)

Here, the biochemical steps included in the signaling pathway will be described briefly; for a detailed description we refer the reader to reviews such as [59,60] and the references therein:

• Upon binding of peptide/MHC to the TCR, the first main step in the TCR-mediated signaling cascade is the activation of the Src-family protein tyrosine kinase p56^lck ^(in the following termed Lck), although the exact mechanism is still unclear. We have included one well accepted mechanism [61], which probably plays a major role but may be combined with others (cf. Figure 10):

 In resting T-cells, the major negative regulator of Lck, the protein tyrosine kinase Csk (C-terminal Src-kinase) is bound via a SH2-domain to the constitutively tyrosine phosphorylated transmembrane adaptor protein PAG (Protein Associated with Glycosphingolipid enriched microdomains) and consequently inhibits membrane-bound Lck by phosphorylating a C-terminal negative regulatory tyrosine residue of the Src kinase.

 Upon ligand binding, PAG is dephosphorylated by a so far unknown protein tyrosine phosphatase, thereby leading to the detachment of Csk from PAG, and hence releasing Lck from the inhibitory effect of Csk. The release of Csk from PAG, together with the activity of the membrane associated tyrosine phosphatase CD45 (which dephosphorylates Lck on the same inhibitory residue that is phosphorylated by Csk), and the concomitant binding of the MHC molecule to the coreceptor CD4, leads to full activation of Lck (see Figure 10).

 However, both CD4 and the TCR can also be stimulated individually, e.g. by using monoclonal antibodies specifically directed at either of the molecules or using cell lines expressing mutated forms of CD4 that cannot bind MHC or cannot transmit signals.

 A regulation of the enzymatic activity of CD45 is not included in the model (basically because it is not yet clear how CD45 is regulated *in vivo*), but, since CD45 is an important regulatory element for T-cells, it is included as an input signal, allowing the analysis of its effect and the performance of CD45 knock-out experiments.

 After a few minutes, PAG is rephosphorylated [62], probably by the Src-kinase Fyn, and subsequently Csk is re-recruited to PAG inhibiting Lck again.

• Activated Lck can phosphorylate another member of the Src-protein kinases, p59^fyn^, in the following termed Fyn (Fyn can probably also be activated in a Lck-independent, TCR-dependent manner [63]). Additionally, Lck phosphorylates the so called ITAMs (Immunoreceptor Tyrosine-based Activation Motifs) that are present in the cytoplasmic domains of the TCR-complex (the latter if the TCR is close to Lck, i.e., if there is a concurrent activation of the TCR). Subsequently, the Syk-family protein tyrosine kinase ZAP70 (Zeta Associated Phosphoprotein of 70 kDa) binds to the phosphorylated ITAMs and, if Lck is active, becomes activated by Lck-mediated tyrosine phosphorylation. Thus, during the initial phase of signal transduction via the TCR three tyrosine kinases become activated in a sequential manner, first Lck and Fyn and then ZAP70. Together these three kinases propagate the TCR-mediated signal by phosphorylating a number of membrane associated and cytosolic signaling proteins.

• Active ZAP70 can phosphorylate LAT (Linker for Activation of T-cells), a second transmembrane adapter protein, at four different tyrosine residues. Subsequently, cytoplasmic signaling molecules containing SH2-domains, including the scaffolding proteins Grb2, Gads, and the lipid kinase PLCγ1 (Phospholipase gamma 1), can bind to phosphorylated LAT. Additionally, Grb2 binds to the nucleotide exchange factor Sos (here we lumped Grb2 and Sos in one activation step), and Gads to the adapter protein SLP76. The latter, upon phosphorylation by ZAP70, can bind to the Tec-family tyrosine kinase Itk. Binding to SLP76 and additional phosphorylation by ZAP70 activates Itk.

• For the activation of PLCγ1, the following conditions have to be fulfilled: PLCγ1 is bound to LAT, SLP76 bound to Gads, ZAP70 is activated (which hence phosphorylates SLP76, allowing PLCγ1 to bind to SLP76), and Itk is active, and hence is able to phosphorylate and thereby to fully activate PLCγ1. Since all these conditions are needed, a logical AND was included in the model (see Figure 10). Rlk, another Lck-dependent Tec-family tyrosine kinase, can also phosphorylate PLCγ1, hence Rlk has a redundant role to Itk with regard to the activation of PLCγ1 [64].

• Activated PLCγ1 hydrolyses phosphatidyl-inositol-4,5 biphosphate (PIP_2_), which is considered an ubiquitous membrane associated phospholipid and is therefore not modeled, thereby generating the second messenger molecules diacyloglycerol (DAG) and inositol trisphosphate (IP3) [59,61].

• IP_3 _mediates calcium flux. Calcium (together with calmodulin) activates the serine phosphatase calcineurin, which dephosphorylates the cytosolic form of the transcription factor NFAT (Nuclear Factor of Activated T-cells). The calcineurin-mediated removal of phosphate groups allows NFAT to translocate to the nucleus and to regulate gene expression.

• The second messenger DAG activates PKCθ and (together with PKCθ[65]) activates the nucleotide exchange factor RasGRP1.

• RasGRP1 and Sos (the latter if it is close to the membrane, that is, if it is bound to LAT by means of Grb2), can activate Ras, which in turn activates the Raf/MEK/ERK MAPK Cascade.

• PKCθ is involved in the activation of JNK, as well as the essential transcription factor NFκB (via phosphorylation and subsequent degradation of the NFκB inhibitor, Iκ B, by the PKCθ-activated Iκ B-kinase, IKK).

• ERK, activated by the Ras/Raf/MEK cascade, activates the transcription factor CRE and (together with JNK) the essential transcription factor AP1.

• The E3 ubiquitin ligase cCbl is important for shutting off TCR-mediated signaling processes by ubiquitination of key proteins, which are subsequently targeted for degradation [66]. One important target of cCbl is ZAP70; upon tyrosine phosphorylation of ZAP70, cCbl binds to ZAP70, leading to ZAP70's ubiquitination and degradation as well as to the downregulation of the TCR.

From these biological facts we constructed a logical hypergraph model, containing 40 nodes and 49 hyperarcs, and implemented it in *CellNetAnalyzer *(Figure 10). The model is summarized in Table 2.

#### Remarks on the logical T-cell activation model

Note that a species can represent different states of a molecule: for example, CD45 refers to the availability of CD45 to act on its substrates (Lck and Fyn), PLCg(bind) refers to PLCγ1 bound to LAT, and PLCg(act) to the active (bound to LAT and phosphorylated) form of PLCγ1. It is also important to realize that several steps can be lumped together or expressed in higher detail; for example, the formation of the complex LAT:Grb2:Sos is considered as one step, but intermediate steps could be considered. This would be reasonable, for example, if Grb2 would have other functions apart from binding Sos. Similarly, the two steps of cCbl's effect (ubiquitination and degradation) are lumped in the hyperarcs pointing to its targets ZAP70 and TCR.

Also note that some of the logical operators could be modeled in a different manner, as in the case of Sos and RasGRP for the activation of Ras (where we prefer an OR since both can independently activate Ras, although both (AND) may be needed for full Ras activation).

Furthermore, our model describes the full activation of the cascade which leads to proliferation; it is known that e.g. stimulation of TCR with antibodies against its CD3 subunits produces a certain activation of the cascade (where probably Fyn overtakes Lck's role [63]) but does not lead to full activation. Therefore, in our model, as an approximation, activated Fyn can phosphorylate the ITAMs of the TCR, but is not able to activate ZAP70. Here a model with more than 2 levels could be envisioned, where activation of Fyn would be enough to produce a weak (level 1) activation of ZAP70 and hence the whole cascade downstream, while full activation via Lck would activate the cascade to a level 2 (full activation).

The model has two extracellular input signals (one for the TCR and one for the coreceptor CD4). Additionally, an input arc for CD45 is included because the regulation of CD45 is not modeled, as described above. Therefore, mathematically speaking, the model contains 3 elements in the input layer. On the other hand, the output layer contains 4 transcription factors (CRE, AP1, NFAT and NFκB).

As explained in the theoretical section, one reasonable way to deal with the effect of negative feedbacks is to consider the different time scales of the processes. Hence, since PAG rephosphorylation takes place after a few minutes [62], and cCbl mediated degradation is an even slower process, we can define several scenarios:

**-**τ = 0, resting-state (no inputs, no feedbacks),

**-**τ = 1, early-events (input(s), no feedbacks), and

**-**τ = 2, mid-time events (input(s), feedbacks). Here, the state of the feedback loops (activation of PAG/Csk by Fyn and recruitment of cCbl to phosphorylated ZAP70) will depend on the state of the respective activators at τ = 1. This can be considered either by fixing manually the state values of cCbl and PAG/Csk for τ = 2 upon inspection at τ = 1 (as was done herein) or by inclusion of a positive self-loop.

We use the term mid-time event since one can also envision a long-term scenario (τ = 3), where slow gene expression mechanisms (not considered here) are active.

#### Analysis of the T-cell signaling cascade

In the interaction graph underlying the hypergraphical model, there are 1158 paths from the input to the output layer and 9 (7 negative and 2 positive) feedbacks loops, which are listed in Table 3. cCbl is involved in most (88%) of the loops, in accordance to its important role in the regulation of the signaling cascade. Not surprisingly, since the only feedback mechanisms included are the effect of cCbl on ZAP70 and TCR and of Fyn on PagCsk, no loop goes downstream of ZAP70, and a suitable minimal cut set attacking all the feedback loops would consist of Fyn and cCbl.

**Table 3:** All negative and positive feedback loops in the T-cell model as determined by CellNetAnalyzer. Negative influences are indicated by "⊣", positive influences are expressed by "→".

1 (negative)	TCRbind → TCRphos → ZAP70 → cCbl ⊣ TCRbind
2 (negative)	TCRbind → Fyn → TCRphos → ZAP70 → cCbl ⊣ TCRbind
3 (negative)	TCRbind ⊣ PAGCsk ⊣ Lck → ZAP70 → cCbl ⊣ TCRbind
4 (negative)	TCRbind ⊣ PAGCsk ⊣ Lck → TCRphos → ZAP70 → cCbl ⊣ TCRbind
5 (negative)	PAGCsk ⊣ Lck → Fyn → PAGCsk
6 (negative)	TCRbind ⊣ PAGCsk ⊣ Lck → Fyn → TCRphos → ZAP70 → cCbl ⊣ TCRbind
7 (negative)	cCbl ⊣ ZAP70 → cCbl
8 (positive)	TCRbind → Fyn → PAGCsk ⊣ Lck → TCRphos → ZAP70 → cCbl ⊣ TCRbind
9 (positive)	TCRbind → Fyn → PAGCsk ⊣ Lck → ZAP70 → cCbl ⊣ TCRbind

**Figure 11:**
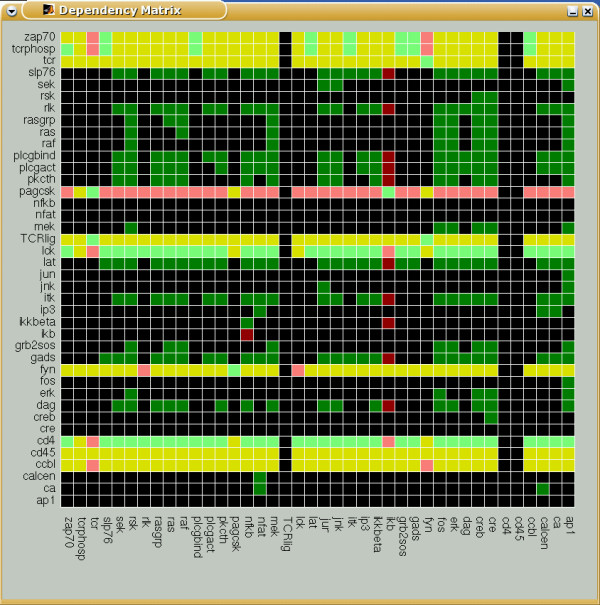
Dependency matrix for the T-cell model. The meaning of the different colors is the same as in Figure 6.

**Figure 12:**
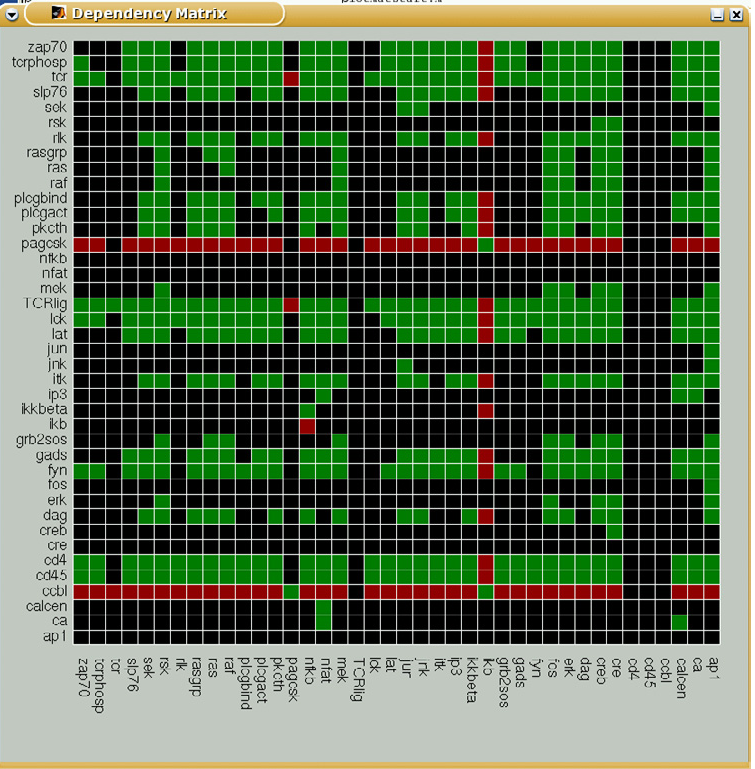
Dependency matrix for the T-cell model for the early event scenario (*τ = 1: the feedback loops are not active)*. The meaning of the different colors is the same as in Figure 6.

We further analyze the interaction graph by computing the dependency matrix (Figure 11). Since downstream of ZAP70 there are only positive connections (except at node IκB), all the elements downstream of ZAP70 are total activators (except of IκB, which is a total inhibitor of NfκB) with respect to the transcription factors in the output layer, that is, they can have only positive effects. Therefore, for these species, a negative intervention via e.g. inhibitors or iRNA would unambiguously lead to a decrease in the activation levels of the transcription factors. For considering the early-events scenario (τ = 1: the feedback loops are not active), we recompute the dependency matrix where the action of Fyn on PAGCsk and of ZAP70 on cCbl is not considered (Figure 12). Then, all inputs (CD45, TCRlig and CD4) are total activators for all species in the output layer. This is not the case when the feedbacks become active (Figure 11): TCRlig and CD45 become then ambivalent factors, i.e. have negative connections to the sink species, whereas CD4 is still an activator but no longer a total one, as it is now connected to a negative feedback loop.

A further analysis of the interaction graph provides that there is no minimal cut set containing only one (essential) species whose removal would interrupt all the positive paths to all the outputs. In fact, all minimal cut sets satisfying this intervention task would contain at least two species, for example MCS1 = {Rlk, ZAP70} and MCS2 = {LAT, PLCg(act)}. The latter examples agree only partially with biological knowledge: removal of MCS1 or MCS2 would indeed prevent the activation of any output, however, from experimental observations one knows that for example LAT alone is essential in TCR signaling [60]. Thus, MCS2 would not be minimal.

Interpreting the *hypergraphical (logical) *model (Figure 10) reveals that, due to several AND connections, the additional removal of PLCg(act) would indeed be redundant because PLCg can anyway not be activated if LAT is removed. This example illustrates the limitations of graph-based methods and we computed therefore the (logical) minimal cut sets from the logical interaction hypergraph revealing that not only LAT, but also ZAP70, Lck, TCR, the ligand for the TCR, TCRphosp, CD4 and CD45 are essential for full T-cell activation. This result is in good agreement with the current knowledge: the T-cell receptor, its ligand, and the ability of the receptor to get phosphorylated are required for T-cell activation; and CD4 (since it binds Lck thus recruiting it to the membrane) and CD45 (which dephosphorylates Lck inhibitory regulatory site) are required for the activation of the essential kinase Lck.

**Figure 13:**
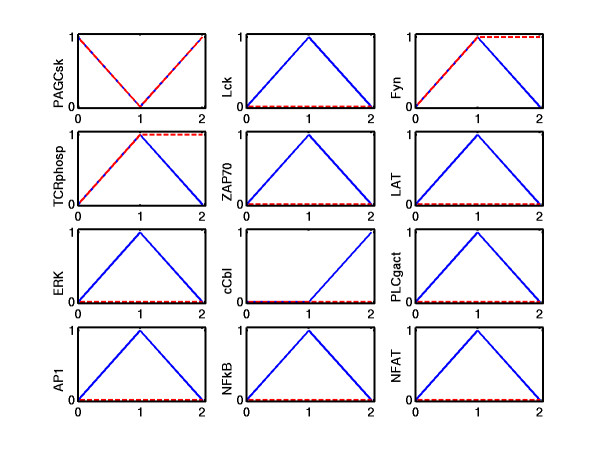
Simulation results of LSS analysis of key elements of the T-cell model using the two time-scales explained in the text. Blue line: upon TCR+CD4+CD45 activation; dashed red line: only TCR+CD45 activation.

Next we performed a logical steady state analysis for the different time scales given above. These simulations provide a rough approximation to the dynamics of the signaling cascade. Figure 10 shows the particular situation in the early-event scenario (τ = 1) as displayed in *CellNetAnalyzer*. Figure 13 summarizes the logical steady state values of important components obtained for the three different time scales. The blue line shows the case for TCR+CD4+CD45 stimulation, whereas the dashed red line represents the case when only TCR+CD45 is stimulated in the input layer. Similar analysis can be performed using different scenarios, for example, in a cell where a certain element has been knocked-out.

## Conclusion

In this contribution we have presented a collection of methods for the functional analysis of the structure of cellular signaling and regulatory networks. As discussed in the theoretical sections, different abstractions and formalisms can be used to encode and analyze the topology of interaction networks. The simplest representations are interaction graphs, which are restricted to one-to-one relationships but do yet capture important functional and causal dependencies in the system under study. We have shown that arguably the most important features of interaction graphs, namely feedback circuits and signaling (or influence) pathways, can systematically be identified by the concept and algorithm of elementary modes known from stoichiometric (metabolic) network analysis. Feedback cycles are mainly responsible for the dynamic behavior of the system, whereas signaling paths reveal network-wide dependencies between species. In some cases, analysis of feedback cycles and signaling paths may allow one to predict unambiguously the qualitative effect upon perturbations of certain species (independently of kinetic parameters and mechanisms). Falsification experiments may then be used to identify missing or incorrect interactions. Knowledge on all the signaling paths also facilitates a systematic identification of optimal intervention strategies. Again, a concept known from metabolic networks, minimal cut sets, can be adapted and employed here. However, inhibitory actions make this kind of analysis more complicated and we therefore generalized the formalism of minimal cut sets leading to minimal intervention sets.

The applicability of tools from metabolic network analysis to interaction graphs relies on the fact that metabolic networks are hypergraphs, which in turn are generalizations of graphs. In our opinion, the importance of hypergraphs in structural analyses of cellular interaction networks has been underestimated. In fact, whenever AND-connections occur in interactions of species, hypergraphical approaches become essential.

Boolean networks describe interaction networks in a more constrained and deterministic manner than interaction graphs, enabling discrete simulations. Herein we have demonstrated that signed directed hypergraphs are capable to represent the logical structure of any Boolean network. The hypergraphical coding of Boolean networks, which relies on the sum-of-product representation of Boolean networks (using only AND, OR and NOT operations), has several advantages: it is rather intuitive, it mostly corresponds to the underlying molecular mechanisms, and it is easy to store and to handle. A hypergraphical representation of a Boolean network also establishes a direct link to the corresponding (underlying) interaction graph which can easily be derived from the hypergraph. Finally, it facilitates a logical signal flow (or steady state) analysis in Boolean networks which, as demonstrated in this report, is useful for studying and predicting the qualitative input-output behavior of signaling networks with respect to a given, possibly incomplete, set of initial state values. This can be achieved here without an explicit enumeration and/or simulation of all possible trajectories.

In general, Boolean networks rely on stronger assumptions and knowledge than interaction graphs and a pure logical description of all interactions is not always possible. We have suggested extensions of the Boolean framework, such as incomplete truth tables of logical operations, to handle these problems.

As pointed out by many authors (e.g. [67-69]) the logical description and analysis of large signaling networks has a strong relationship to electrical circuit analysis; however, there still seems to be a large potential in employing theoretical and software tools from electrical engineering and Boolean logic for investigating interaction networks. Signal flow analysis as introduced herein might be another step in this direction.

Describing signal and mass flows equivalently as interactions, as done herein, offers high flexibility and enables one to integrate several types of cellular networks (such as metabolic, signalling or regulatory ones) into one framework. However, the higher level of abstraction comes with the price that some molecular mechanisms are not always precisely represented, as, for instance, the stoichiometric coefficients in mass flows.

The potential of the introduced methods were demonstrated on a model of a small part of the signaling machinery of T-cells. The size and complexity of the model was chosen so that the methods could be tested on a case study of real size and complexity, while at the same time the results could be (at least in part) intuitively understood and proofed. If enough information is available, similar models could be set up for any other signaling network.

Certainly, these tools will be especially useful in larger interaction networks. Our current and future work aims to expand and subsequently analyse the T-cell model, with hopes that further understanding of this complex network can improve current knowledge about important illnesses, such as autoimmune diseases and leukemia. This is certainly a challenging task, but the potential described here makes it a worthy endeavour.

## Availability and requirements

For academic purposes,*CellNetAnalyzer *can be obtained for free via the website



Note that *CellNetAnalyzer *requires MATLAB^® ^version 6.1 or higher.

## List of abbreviations

LIH: logical interaction hypergraph

LSS(s): logical steady state(s)

MCS(s): minimal cut set(s)

MIS(s): minimal intervention set(s)

## Authors' contributions

SK elaborated the framework and the methods for studying interaction graphs and logical interaction hypergraphs and implemented algorithms in *CellNetAnalyzer*. JSR mainly constructed the logical model of T-cell signalling and he also contributed to the methods' development. JL and LS assisted in the construction of the T-cell model. EDG initiated the project on methods for structural analysis of signaling networks. SK and JSR prepared the manuscript jointly. All authors have read and accepted the manuscript.
